# Comparison of the impact of two key fungal signalling pathways on *Zymoseptoria tritici* infection reveals divergent contribution to invasive growth through distinct regulation of infection‐associated genes

**DOI:** 10.1111/mpp.13365

**Published:** 2023-06-12

**Authors:** Harry T. Child, Michael J. Deeks, Jason J. Rudd, Steven Bates

**Affiliations:** ^1^ Department of Biosciences University of Exeter Exeter UK; ^2^ Department of Protecting Crops and the Environment Rothamsted Research Harpenden UK

**Keywords:** cAMP signalling, cell wall integrity, effectors, plant pathogen, virulence, wheat, *Zymoseptoria tritici*

## Abstract

The lifecycle of *Zymoseptoria tritici* requires a carefully regulated asymptomatic phase within the wheat leaf following penetration of the mesophyll via stomata. Here we compare the roles in this process of two key fungal signalling pathways, mutants of which were identified through forward genetics due to their avirulence on wheat. Whole‐genome resequencing of avirulent *Z. tritici* T‐DNA transformants identified disruptive mutations in *ZtBCK1* from the kinase cascade of the cell wall integrity (CWI) pathway, and the adenylate cyclase gene *ZtCYR1*. Targeted deletion of these genes abolished the pathogenicity of the fungus and led to similar in vitro phenotypes to those associated with disruption of putative downstream kinases, both supporting previous studies and confirming the importance of these pathways in virulence. RNA sequencing was used to investigate the effect of *ZtBCK1* and *ZtCYR1* deletion on gene expression in both the pathogen and host during infection. *ZtBCK1* was found to be required for the adaptation to the host environment, controlling expression of infection‐associated secreted proteins, including known virulence factors. Meanwhile, *ZtCYR1* is implicated in controlling the switch to necrotrophy, regulating expression of effectors associated with this transition. This represents the first study to compare the influence of CWI and cAMP signalling on in planta transcription of a fungal plant pathogen, providing insights into their differential regulation of candidate effectors during invasive growth.

## Graphical abstract

The importance of *Zymoseptoria tritici* cell wall integrity and cAMP signalling on virulence and in planta gene expression was compared, demonstrating their differential regulation of candidate effectors during invasive growth.

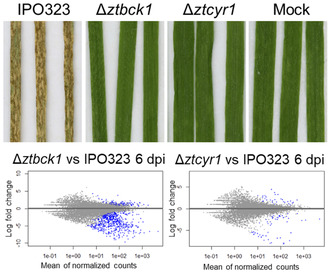

## 1 INTRODUCTION

Signalling cascades are integral for the ability of cells to adapt to their environment, enabling them to couple the recognition of external cues at the surface with appropriate intracellular processes, such as regulation of gene expression and protein activity. For fungal pathogens of plants, this involves regulating crucial changes in morphology and expression of virulence factors in response to the host environment. This is essential for controlling developmental transitions during infection, involving host surface recognition and penetration, invasive growth, and differentiation of reproductive structures. It is also important for responding to the stresses imposed by the host environment, such as nutrient deprivation and the plant immune response.

The cell wall integrity (CWI) pathway is a conserved fungal signalling cascade that regulates remodelling of the cell wall (Levin, 2011). This pathway transduces cell wall perturbation signals recognized by mechanosensors at the cell surface to the conserved mitogen‐activated protein kinases (MAPK) Slt2. Modulation of cell wall composition is required for fungal growth and morphological transitions (Cabib & Arroyo, 2013; Riquelme et al., 2018), as well as responses to various stresses, including those encountered in the host environment (Geoghegan et al., 2017; Hopke et al., 2018). Furthermore, fungal pathogens adapt their cell wall composition to avoid host detection, as both chitin and β‐1,3‐glucan are recognized as pathogen‐associated molecular patterns (PAMPs) by host immune systems (Ballou et al., 2016; El Gueddari et al., 2002; Fujikawa et al., 2012).

The CWI pathway has been found to play a crucial role in the virulence of plant fungal pathogens (Turrà et al., 2014), with functions in host penetration (Kojima et al., 2002; Rui & Hahn, 2007; Xu et al., 1998) and invasive growth (Joubert et al., 2011; Mehrabi et al., 2006; Mey et al., 2002; Rui & Hahn, 2007). The latter function has been attributed to the role of the CWI pathway in withstanding host defences, as *Slt2* deletion was shown to increase sensitivity to hydrolytic enzymes (Mey et al., 2002; Rui & Hahn, 2007; Xu et al., 1998), as well as plant‐derived antimicrobial compounds (Joubert et al., 2011; Ramamoorthy et al., 2007).

Another signalling cascade that has emerged as crucial for the virulence of fungal plant pathogens is the cyclic adenosine monophosphate (cAMP)‐dependent protein kinase A (PKA) pathway (Turrà et al., 2014). In *Saccharomyces cerevisiae*, cAMP generated by the adenylate cyclase enzyme binds to the regulatory subunits (encoded by *BCY1*) of the PKA complex, allowing the catalytic subunits (encoded by *TPK1‐3*) to dissociate and phosphorylate downstream targets. This pathway functions in nutrient sensing in yeast to regulate carbohydrate metabolism, the cell cycle, growth, and development (Zaman et al., 2008). However, the cAMP‐PKA pathway is known to regulate an extremely diverse range of fungal processes, including morphological transitions (D'Souza & Heitman, 2001), sexual reproduction (Hu et al., 2014), secondary metabolism (Studt et al., 2013), and responses to diverse nutritional signals (Caza & Kronstad, 2019). cAMP‐PKA signalling is required for development of host penetration structures in many plant‐pathogenic fungi, including *Magnaporthe oryzae* (Choi & Dean, 1997; Xu et al., 1997) and *Fusarium graminearum* (Bormann et al., 2014). Furthermore, there is also evidence for the role of this pathway in post‐penetration invasive growth, with strains lacking cAMP‐PKA components unable to cause disease following inoculation into experimental wounded leaf tissue (Li et al., 2017; Yamauchi et al., 2004).

The ascomycete pathogen *Zymoseptoria tritici* causes the most economically important disease of wheat in Europe (Jørgensen et al., 2014). Colonization of wheat by *Z. tritici* involves complex regulation of morphological development and the molecular interaction with the host, requiring dramatic shifts in gene expression across different infection stages (Rudd et al., 2015). Initial colonization involves hyphal germination on the leaf surface and invasion of the host stomata (Kema et al., 1996). *Z. tritici* then grows asymptomatically in the apoplast whilst suppressing the host immune response through expression of secreted effectors, including essential repression of chitin recognition by the LysM effectors (Marshall et al., 2011; Rudd et al., 2015). Finally, *Z. tritici* switches to necrotrophic growth, instigating host programmed cell death through up‐regulation of a distinct set of effectors and acquiring nutrients from dying host tissue through expression of secreted enzymes and membrane transporters (Rudd et al., 2015).

The CWI pathway has been proposed to control *Z. tritici* resistance to apoplastic wheat defence compounds during asymptomatic invasion, as Δ*mgslt2* mutants were unable to colonize the mesophyll tissue following stomatal penetration and showed increased sensitivity to cell wall‐degrading enzymes (Mehrabi et al., 2006). Furthermore, deletion of the PKA catalytic subunit *MgTpk2* led to delayed symptom development and abolished pycnidia formation, despite successful host penetration and invasive growth, suggesting its involvement in triggering the switch to necrotrophy and asexual development (Mehrabi & Kema, 2006). However, despite these investigations, links between these regulatory pathways and the transcription of genes involved in host manipulation by *Z. tritici* remain to be elucidated.

In this study, forward genetic investigation of avirulent T‐DNA insertion transformants identified disruptions to *Z. tritici* genes encoding adenylate cyclase and the CWI MAPK cascade enzyme BCK1. Targeted deletion of these genes was used to confirm that loss of their functions led to abolishment of pathogenicity. Despite showing defects during in vitro vegetative growth, deletion strains were able to penetrate host stomata, supporting previous evidence for the role of the CWI and cAMP‐PKA pathways during infection post‐penetration. Finally, RNA sequencing was used to characterize transcription in both *Z. tritici* and wheat during infection by these strains to investigate the enigmatic in planta functions of these pathways. This revealed different suites of candidate effectors involved in invasive growth regulated by these two pathways and provided insight into how this differential regulation impacts the host defence response.

## 2 RESULTS

### 2.1 Nonsynonymous SNP identified in *ZtCYR1* gene of avirulent T‐DNA insertion strains

Two transformants with severely reduced pathogenicity, designated L2 and C5, were identified during in planta phenotyping of targeted deletion mutants that aimed to inactivate the genes ZtritIPO323_04g10737 (Mycgr3G43288) and ZtritIPO323_04g13543 (Mycgr3G18212), encoding putative secreted lipase (*ZtL2*) and cutinase (*ZtCUT5*) proteins, respectively. L2 and C5 did not cause necrosis or develop pycnidia, although patches of mild chlorosis were observed at 21 days postinoculation (dpi) (Figure S1a). This phenotype was contrary to two PCR‐validated independent deletion mutants in these target genes, which displayed wild‐type virulence (Figure S1a). Whole‐genome resequencing of C5 and L2 found no evidence for ectopic insertion of transformed T‐DNA (Figure S2a,b), suggesting that avirulence in these strains was caused by random mutation(s). Variant calling found these strains to share 15 polymorphisms compared to the isogenic IPO323 strain (Table S2), suggesting that they originate from the same genotype in the background population. These included two within the coding regions of genes; a 3 bp deletion was identified in the first exon of *ZtAGO1* (ZtritIPO323_04g08085/Mycgr3G38035), causing a P76del deletion, and a nonsynonymous single‐nucleotide polymorphism (SNP) in the second exon of the *Z. tritici* homologue of adenylate cyclase enzyme *MgCYR1* (ZtritIPO323_04g11209/Mycgr3G86659), hereafter *ZtCYR1* (Table S2). The latter substitution causes a missense mutation of E1663K at a predicted nucleotidyl binding site in the cyclase domain (IPR001054) of ZtCYR1 (Figure S2c). Previous studies have found *ZtAGO1* to have a mild or no contribution to virulence (Habig et al., 2021; Kettles et al., 2019), while other components of the cAMP‐PKA pathway are known to contribute to the virulence of *Z. tritici* (Mehrabi et al., 2009; Mehrabi & Kema, 2006). Considering this, disruption of *ZtCYR1* was hypothesized to cause the loss of virulence in the strains C5 and L2, prompting further functional characterization of this gene.

### 2.2 Ectopic T‐DNA insertion causes disruption of *ZtBCK1* gene and promotor region

A third avirulent T‐DNA insertion mutant, referred to as T21, was identified during screening of transformants generated for targeted deletion of ZtritIPO323_04g07737 (Mycgr3G108617), encoding a triacylglycerol lipase (*ZtTGL1*). T21 was identified as having a defect in melanization during subculturing. Unlike three independent Δ*zttgl1* mutants, which displayed wild‐type virulence, T21 was avirulent (Figure S1b). Whole‐genome resequencing confirmed the absence of T‐DNA insertion at the ZtTGL1 locus but identified insertion of the full T‐DNA sequence at a locus on chromosome 11 (Figure S3). The ectopic insertion site lies 133 bp upstream of the gene ZtritIPO323_04g03043 (Figure S3), hereafter *ZtBCK1*, encoding the *Z. tritici* homologue yeast mitogen‐activated protein kinase kinase kinase regulating Slt2 in the CWI pathway (Lee et al., 1992). Furthermore, variant calling identified a 12 bp deletion (chr11:240,721‐240,732) in the coding sequence of *ZtBCK1*, causing a four amino acid deletion G6_R9del (Table S2 and Figure S3) outside any predicted conserved domains. Variant calling also identified a nonsynonymous SNP in the second exon of ZtritIPO323_04t11145 (Mycgr3G60318), encoding a putative major facilitator superfamily (MFS) transporter (Table S2). However, considering the previous evidence for the virulence‐related function of the CWI pathway in *Z. tritici* (Mehrabi et al., 2006), the disruption of *ZtBCK1* was chosen for further investigation into the cause of avirulence in T21.

### 2.3 Targeted deletion of *ZtBCK1* and *ZtCYR1* abolishes virulence in *Z. tritici*

**FIGURE 1:**
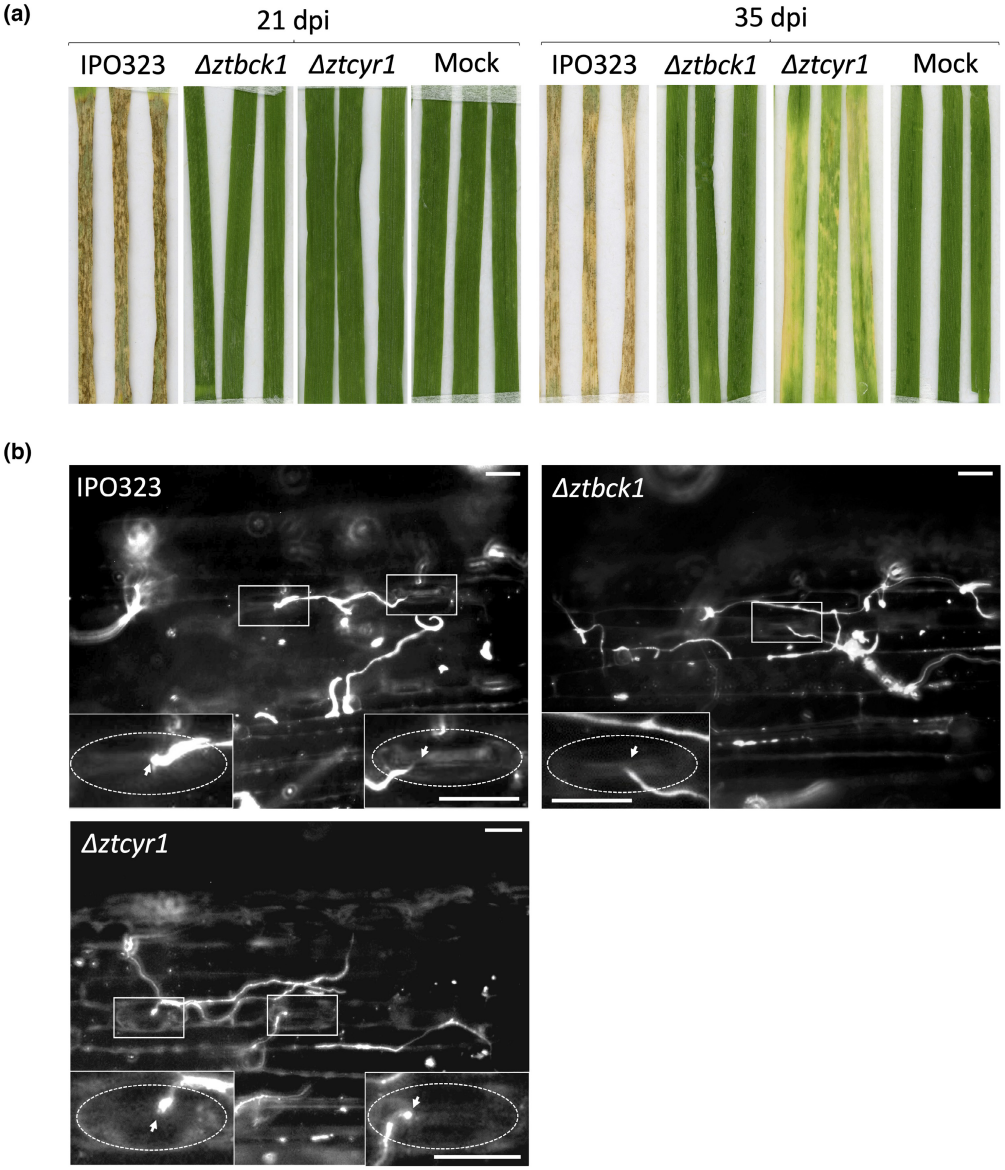
Targeted deletion of *ZtBCK1* and *ZtCYR1* leads to abolition of *Zymoseptoria tritici* virulence. (a) Disease symptoms on wheat leaves after 21 and 35 days postinoculation (dpi) by Δ*ztcyr1* and Δ*ztbck1* strains compared to wild‐type IPO323. (b) Epiphytic *Z. tritici* hyphae stained with calcofluor white on the leaf surface 3 dpi. Magnified images detailing hypha–stomata interactions (arrows), with guard cells indicated by dotted oval. Scale bars = 40 μm.

To investigate the function of *ZtBCK1* and *ZtCYR1* in *Z. tritici* development and virulence, three independently constructed null mutants were generated for both genes (Figure S4a,b), and these were shown to display comparable phenotypes both in vitro and in planta. The resulting Δ*ztcyr1* and Δ*ztbck1* strains showed equivalent attenuation of virulence to that displayed by strains L2/C5 and T21, with all deletion strains unable to induce necrosis or develop pycnidia at 21 dpi (Figure 1a). Although the mild chlorosis caused by L2 and C5 was not observed on Δ*ztcyr1*‐infected leaves at 21 dpi, continuation of infection to 35 dpi led to the appearance of chlorosis on these leaves (Figure 1a). Chlorosis was not observed on mock‐inoculated or Δ*ztbck1‐*infected leaves, confirming that this was not the result of environmental conditions or senescence (Figure 1a). These findings provide supporting evidence that the avirulence of L2/C5 and T21 is probably caused by disruption of *ZtCYR1* and *ZtBCK1*, respectively.

Microscopic observations revealed no difference in stomatal interactions between both Δ*ztcyr1* and Δ*ztbck1* strains and wild‐type IPO323 (Figure 1b). Spores of both mutants germinated on the leaf surface to produce epiphytic hyphae, and numerous stomatal interactions were observed for all strains at 3 dpi (Figure 1b). These findings establish that avirulence in these strains is not caused by disruption of the morphological switch to hyphal growth, sensory perception of the host surface or recognition of stomatal apertures. Instead, this suggests that signalling via ZtCYR1 and ZtBCK1 is required during infection stages after host penetration.

### 2.4 Deletion of *ZtBCK1* impacts vegetative growth and the cell wall stress response

**FIGURE 2:**
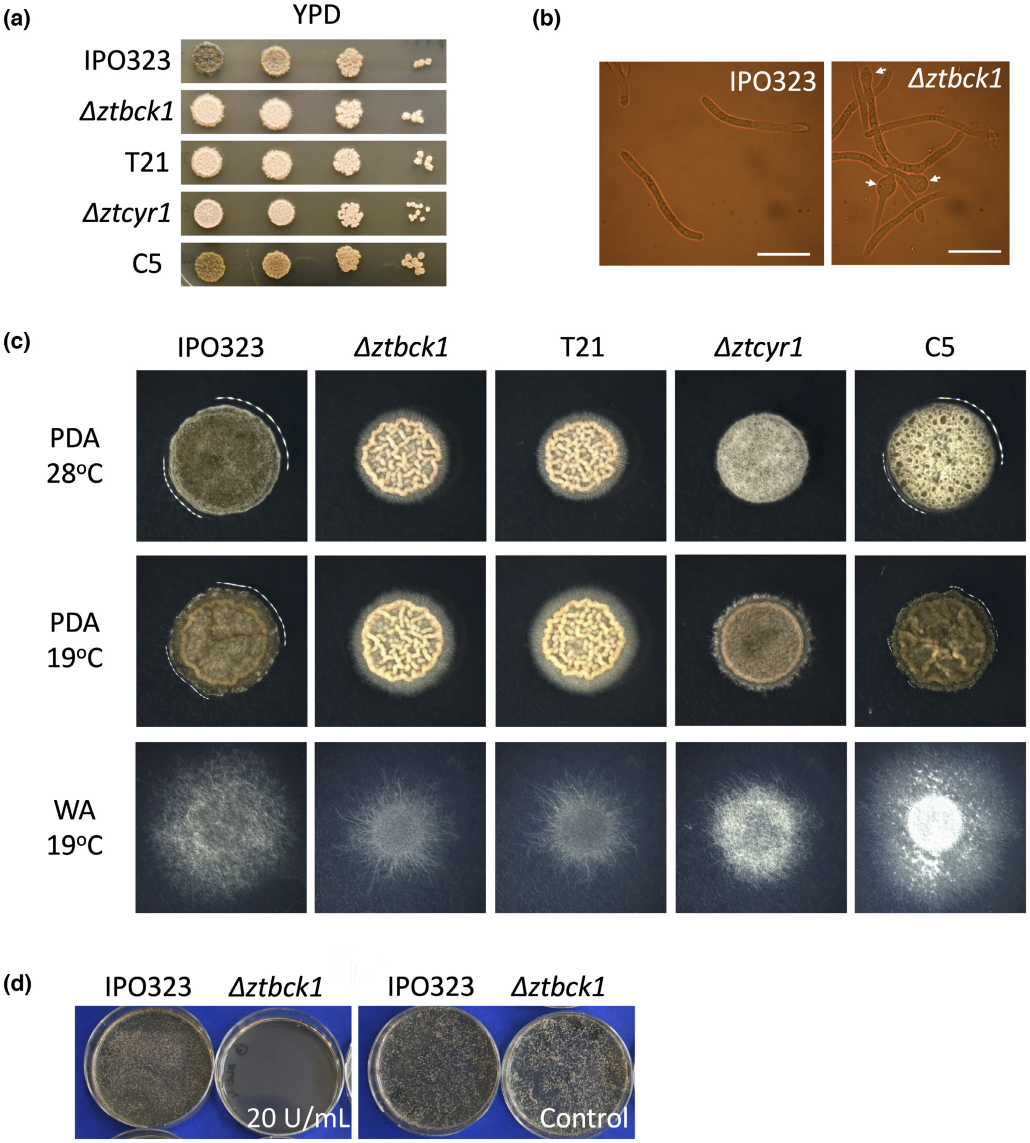
Vegetative growth phenotypes of *ZtBCK1* and *ZtCYR1* deletion strains. (a) *Zymoseptoria tritici* grown for 10 days on yeast peptone dextrose (YPD) at 19°C, 5‐μL droplets of a 10‐fold serial dilution starting at a concentration of 5 × 10^6^ spores/mL. (b) Blastospores grown on YPD for 6 days, with appearance of swollen cells (arrows) in Δ*ztbck1*. Scale bar = 20 μm. (c) Growth on potato dextrose agar (PDA) at 28 and 19°C for 7 days and radial hyphal growth on water agar (WA) at 19°C for 14 days. (d) *Z. tritici* cell suspensions exposed 20 U/mL of lyticase enzyme from *Arthrobacter luteus* for 2 h at 25°C before being cultured on YPD agar for 5 days at 19°C, showing loss of viability of Δ*ztbck1* strains at lower enzyme concentrations than the wild type IPO323.

Considering previous reports of the CWI pathway influencing both yeast‐like and hyphal growth of *Z. tritici* (Mehrabi et al., 2006), the vegetative growth characteristics of Δ*ztbck1* strains were investigated. While the growth rate by blastosporulation on yeast peptone dextrose (YPD) agar was consistent between Δ*ztbck1* strains and IPO323, a defect in melanization was seen in Δ*ztbck1* mutants after a prolonged growth period (Figure 2a). Furthermore, swollen cells were observed regularly in the blastospores of Δ*ztbck1* strains after 6 days of growth on YPD, which were absent in IPO323 cells under the same conditions (Figure 2b). Although germination efficiency and early colony formation on water agar (WA) was unchanged in Δ*ztbck1* strains, their growth rate was reduced over time as radial growth continued, resulting in smaller colonies than the wild type after 14 days (Figure 2c). In addition, while the wild type developed abundant aerial hyphae at 28°C on potato dextrose agar (PDA), Δ*ztbck1* strains displayed equivalent growth by blastosporulation at 19 and 28°C (Figure 2c). These findings suggest that the *Z. tritici* CWI pathway is involved in regulating the morphological switch to hyphal growth in response to heat stress, as well as in prolonged hyphal growth under starvation. Finally, the in vitro phenotypes of Δ*ztbck1* were entirely consistent with those of T21, providing further evidence for *ZtBCK1* disruption being the sole genetic cause of the phenotypes of this strain.

The sensitivity of wild‐type and Δ*ztbck1* strains to cell wall stress was assessed by exposure to enzyme mixtures possessing β‐1,3‐glucanase activity. The wild type was able to maintain viability after exposure to a higher concentration of lyticase enzymes than Δ*ztbck1*, providing evidence that deletion of *ZtBCK1* leads to enhanced sensitivity to cell wall stress (Figure 2d). To investigate whether this effect was specific to disruption of β‐1,3‐glucan, the sensitivity of Δ*ztbck1* to calcofluor white, which disrupts chitin synthesis, and caspofungin, which inhibits β‐1,3‐glucan synthesis, was assessed. While Δ*ztbck1* strains displayed no change in sensitivity to calcofluor white, they were found to have elevated sensitivity to caspofungin (Figure S5a). Together, these results suggest that the *ZtBCK1* is specifically involved in regulation of the *Z. tritici* response to perturbation of β‐1,3‐glucan integrity. Furthermore, Δ*ztcyr1* strains, but not strain C5, also displayed increased sensitivity to caspofungin (Figure S5a), suggesting that cAMP signalling may also play a role in this response.

### 2.5 Deletion of *ZtCYR1* impairs melanization and increases osmosensitivity

Vegetative growth phenotypes of Δ*ztcyr1* strains were also investigated for comparison with previously characterized other components of the PKA signalling pathway (Mehrabi et al., 2009; Mehrabi & Kema, 2006). The growth rate by blastosporulation on YPD was indistinguishable from the wild type (Figure 2a). However, melanization was impaired in Δ*ztcyr1* when grown on YPD and PDA at 19°C in the dark (Figure 2a,c). Similarly, melanization of aerial hyphae was not observed in Δ*ztcyr1* strains when grown on PDA at 28°C. Furthermore, while the germination efficiency of Δ*ztcyr1* strains on WA was unchanged from the wild type, subsequent hyphal growth proceeded at a much slower rate in these mutants (Figure 2c). Interestingly, the disruption of melanization and hyphal growth rate was not observed in the C5 strain. Finally, sensitivity to high osmolarity of the growth media was increased in the C5 and Δ*ztcyr1* strains, which displayed a reduced growth rate compared to the wild type on PDA amended with 1.5 M sorbitol (Figure S5b). These findings suggest that *ZtCYR1* is involved in the regulation of melanization, hyphal growth, and the osmotic stress responses, but that the phenotype potentially caused by the point mutation in *ZtCYR1* in the C5 strain is distinct from that resulting from deletion of the whole coding sequence.

### 2.6 Deletion of *ZtBCK1* and *ZtCYR1* impacts in planta growth and gene transcription

Considering the observation of successful epiphytic growth and stomatal interactions during infection by Δ*ztcyr1* and Δ*ztbck1* strains, we hypothesized that avirulence in these strains was caused by defective regulation of genes and processes required for intercellular colonization of the leaf tissue. To investigate this, transcriptome profiling of both *Z. tritici* and wheat during infection by the wild‐type IPO323, Δ*ztcyr1*, and Δ*ztbck1* was carried out by RNA sequencing. Infected leaf samples were taken at 6 dpi to assess differences in gene expression during the late symptomless phase of wild‐type infection, when stomatal penetration by most epiphytic hyphae has occurred and invasive growth is being established in the mesophyll. Samples were also taken at 9 dpi at the start of the transition to necrotrophic growth by the wild‐type IPO323, which has been shown previously to coincide with dramatic transcriptional changes in both the pathogen and host (Rudd et al., 2015). It must be noted that leaf samples infected by all *Z. tritici* strains remained asymptomatic at 9 dpi before the wild type began to induce chlorosis between 10 and 12 dpi.

**FIGURE 3:**
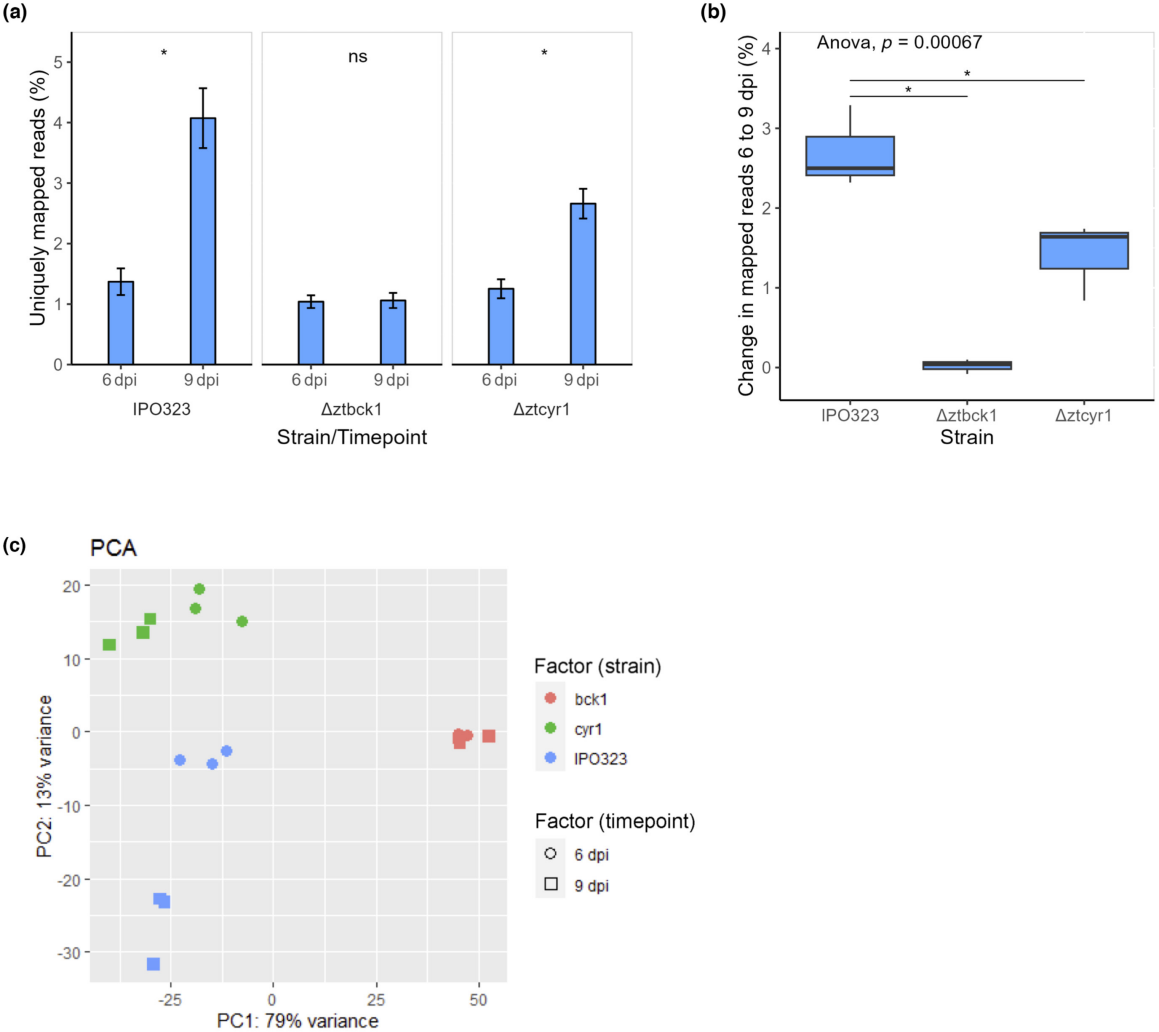
In planta growth and global gene transcription are impacted by deletion of *ZtBCK1* and *ZtCYR1*. (a) Percentage of RNA sequencing reads uniquely mapped to the *Zymoseptoria tritici* genome across infection time points in each strain, as an indication of in planta fungal biomass. Mean values across each repeated sample are plotted along with the standard error. Statistically significant differences between percentages across time points in each strain from paired *t* test are indicated (*p < 0.05). (b) Change in the percentage of uniquely mapped reads between 6 and 9 days postinoculation (dpi) in each experimental repeat grouped by strain, as an indication of growth between these time points. *p* value from one‐way analysis of variance is given and statistically significant differences from pairwise *t* test comparison of mutants to IPO323 are indicated (*p < 0.05). (c) Principal component analysis (PCA) of global normalized gene expression data.

The progress of leaf colonization by each strain between the sampled time points was assessed quantitatively through the relative abundance of *Z. tritici* reads as a measure of fungal biomass. No significant difference in the proportion of *Z. tritici* transcripts was identified between the strains at 6 dpi, suggesting that fungal growth rate up to this point of infection was largely equivalent (Figure 3a). IPO323 displayed significant increases in the proportion of reads mapped to the *Z. tritici* genome at 9 dpi (Figure 3a). This is consistent with previous reports of measurable increases in fungal biomass as *Z. tritici* transitions to necrotrophy, changes which are difficult to detect during early symptomless growth (Keon et al., 2007; Palma‐Guerrero et al., 2016; Rudd et al., 2015). This provides evidence that the infection time points where samples were taken effectively encompass this transition in the infection stage.

In contrast to the wild type, no significant increase in the percentage of reads mapped to the *Z. tritici* genome was identified between 6 and 9 dpi for the Δ*ztbck1* samples, indicating that there was no significant increase in the relative biomass of this strain in infected leaf tissue (Figure 3a). This suggests that colonization of the wheat leaf by Δ*ztbck1* is inhibited during the symptomless phase of infection. Furthermore, while Δ*ztcyr1* samples did display an increase in *Z. tritici* mapped reads between 6 and 9 dpi (Figure 3a), the magnitude of this increase was significantly greater in IPO323 than in Δ*ztcyr1‐*infected leaves (Figure 3b). This suggests that the increase in fungal biomass between these time points was less pronounced for the Δ*ztcyr1* strain.

Principal component analysis (PCA) using global normalized gene expression data revealed that the *Z. tritici* transcriptome of each sample could be most easily distinguished by the strain used for infection (Figure 3c). The first principal component (PC1) encompasses most of the variation in gene expression between Δ*ztbck1* and both Δ*ztcyr1* and IPO323, explaining 76% of the variation in the dataset (Figure 3c). The second principal component (PC2), which explains 16% of the variation in gene expression, appears to comprise the majority of the variation between Δ*ztcyr1* and IPO323, which is accentuated by the progression of infection to 9 dpi (Figure 3c). Although global gene expression in Δ*ztcyr1* does appear to change between the time points, with clear grouping of Δ*ztcyr1* 6 and 9 dpi samples, this change is not as dramatic as the wild type (Figure 3c). These results suggest that divergence in global gene expression between IPO323 and Δ*ztcyr1* increases as the wild type transitions to necrotrophic growth. This led to the hypothesis that disruption of cAMP signalling impairs the ability of *Z. tritici* to undergo this necrotrophic switch. Furthermore, Δ*ztbck1* shows no variation between the sampled time points (Figure 3c), which supports the hypothesis that colonization by Δ*ztbck1* does not progress beyond the initial invasion of the wheat leaf.

### 2.7 *ZtBCK1* and *ZtCYR1* regulate distinct sets of putative secreted proteins and candidate effectors in planta

**FIGURE 4:**
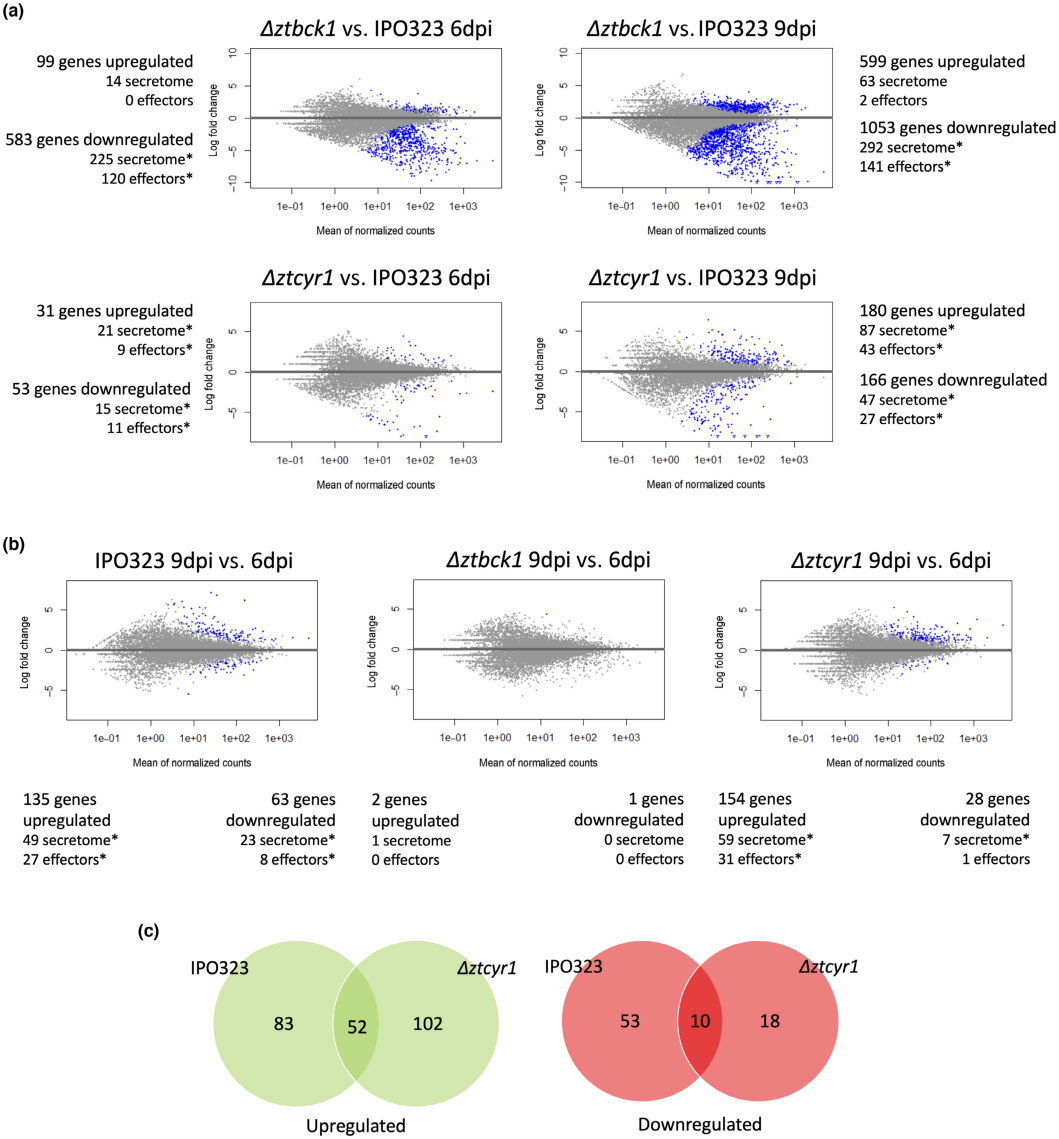
Δ*ztbck1* shows widespread down‐regulation of secreted effectors and Δ*ztcyr1* gene expression diverges from IPO323 as infection progresses. MA plots, of the log fold change in expression versus expression level (mean of normalized counts) are shown, displaying differentially expressed genes (blue points) between (a) mutant strains (Δ*ztbck1* and Δ*ztcyr1*) and IPO323 at 6 and 9 days postinoculation (dpi), and (b) between 9 and 6 dpi in IPO323 (left), Δ*ztbck1* (middle), and Δ*ztcyr1* (right). The number of differentially expressed genes in each comparison are detailed, as well as the number of secretome and predicted effector genes in those differentially expressed sets. Asterisks indicate significant enrichment of these gene categories amongst differentially expressed gene sets determined using Fisher's exact tests (*p* < 0.01). (c) Comparison of genes found to be differentially expressed between 6 and 9 dpi in IPO323 and Δ*ztcyr1*.

Differential expression analysis led to the identification of 682 differentially expressed genes (DEGs) in Δ*ztbck1* at 6 dpi, which increased to 1652 DEGs at 9 dpi compared to the expression profile in IPO323 (Figure 4a and Table S3). Furthermore, only three genes were found to be differentially expressed between the sampling time points in Δ*ztbck1*, compared to 198 DEGs identified between 6 and 9 dpi in IPO323 (Figure 4b and Table S3). These results suggest that not only is gene expression in the Δ*ztbck1* mutant significantly different to the wild type during early infection, but that it also displays none of the transcriptional changes associated with the transition to necrotrophy.

Contrastingly, differential expression analysis found there to be 84 DEGs between Δ*ztcyr1* and IPO323 at 6 dpi, which increased to 346 DEGs at 9 dpi (Figure 4a and Table S3). Furthermore, a set of 182 genes was found to be differentially expressed between 6 and 9 dpi in Δ*ztcyr1* (Figure 4b and Table S3). Although this set showed some similarity to the genes found to be differentially expressed between the time points in IPO323, the majority (66%) were distinct (Figure 4c). These results support those from PCA in suggesting that while global transcription is similar between Δ*ztcyr1* and IPO323 at 6 dpi, the progression of infection to 9 dpi leads to divergence in gene expression between these strains.

**FIGURE 5:**
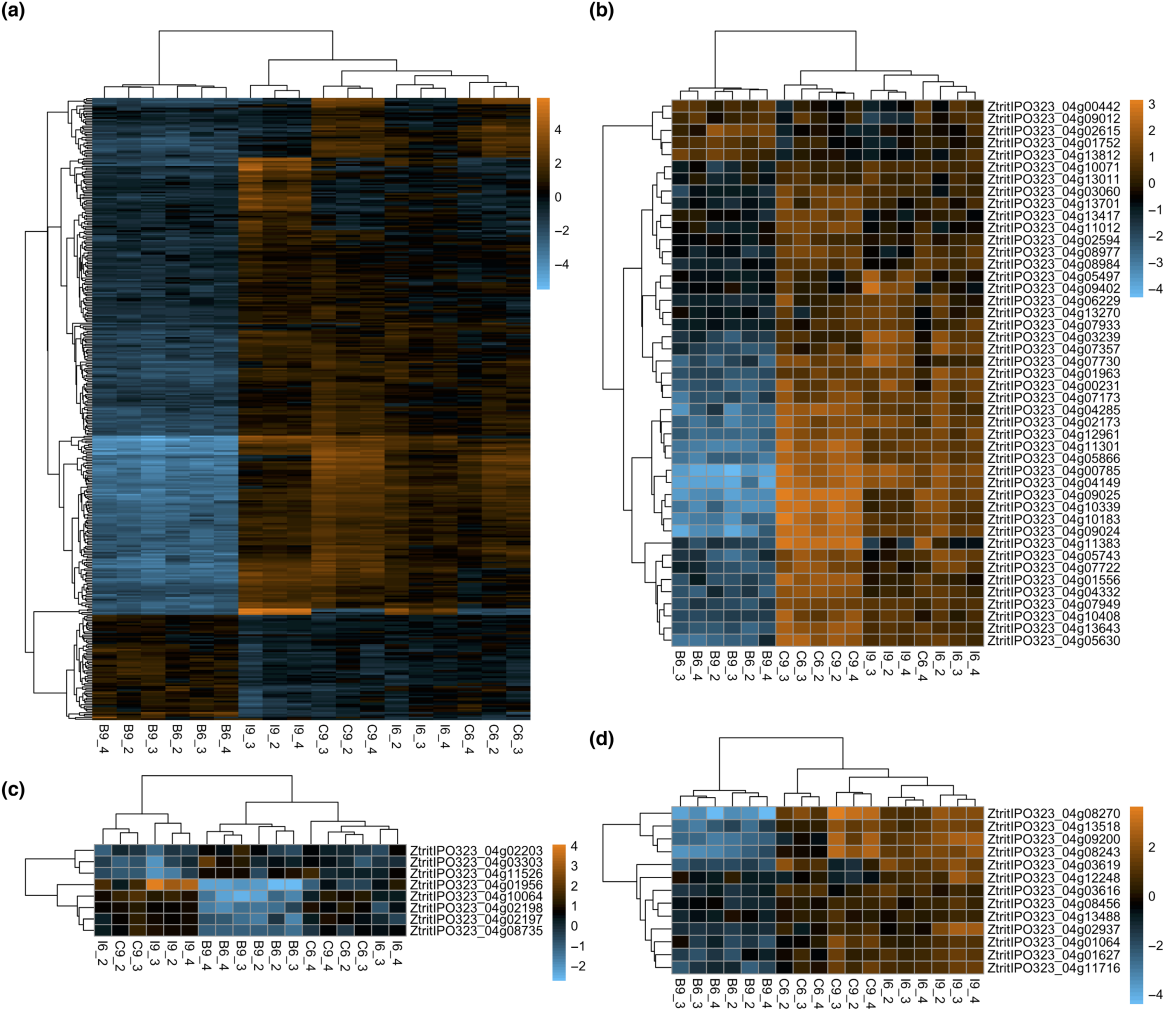
Differential expression of secreted proteins in Δ*ztbck1* and Δ*ztcyr1*. Heat maps displaying log‐fold change in normalized expression values relative to the mean normalized expression for differentially expressed (a) secretome genes, (b) proteases, (c) peroxidase/superoxide dismutases, and (d) plant cell wall‐degrading enzymes. Column labels (e.g., B6_2) indicate the sample strain (B, Δ*ztbck1*; C, Δ*ztcyr1*; I, IPO323), time point (6 and 9 days postinocuation [dpi]), and replicate (2, 3, and 4).

Given the importance of secreted proteins in the molecular interaction between *Z. tritici* and wheat, the differential expression of genes within the recently updated *Z. tritici* secretome was investigated (King et al., 2017). The secretome was defined as those genes within the Rothamsted genome annotation that contain a predicted signal peptide and do not contain a predicted transmembrane domain. Δ*ztbck1* displayed widespread down‐regulation of genes within the secretome compared to IPO323 at both time points during infection (Figures 4a and 5a, and Table S3). Secretome genes were significantly enriched in the down‐regulated genes identified in Δ*ztbck1* at both 6 and 9 dpi, making up 38.6% and 27.7% in these sets, respectively (Figure 4a). Functional annotation based on InterPro domains revealed that these down‐regulated secreted proteins include many with enzymatic activities that have been shown to be induced during wild‐type *Z. tritici* infection (Palma‐Guerrero et al., 2016; Rudd et al., 2015). This includes a host of secreted proteases, plant cell wall‐degrading enzymes, peroxidases, and superoxide dismutases (Figure 5b–d, and Table S3). Overall, these results suggest that Δ*ztbck1* is unable to induce the transcription of a broad range of infection‐related secreted proteins in response to colonization of the host environment.

**FIGURE 6:**
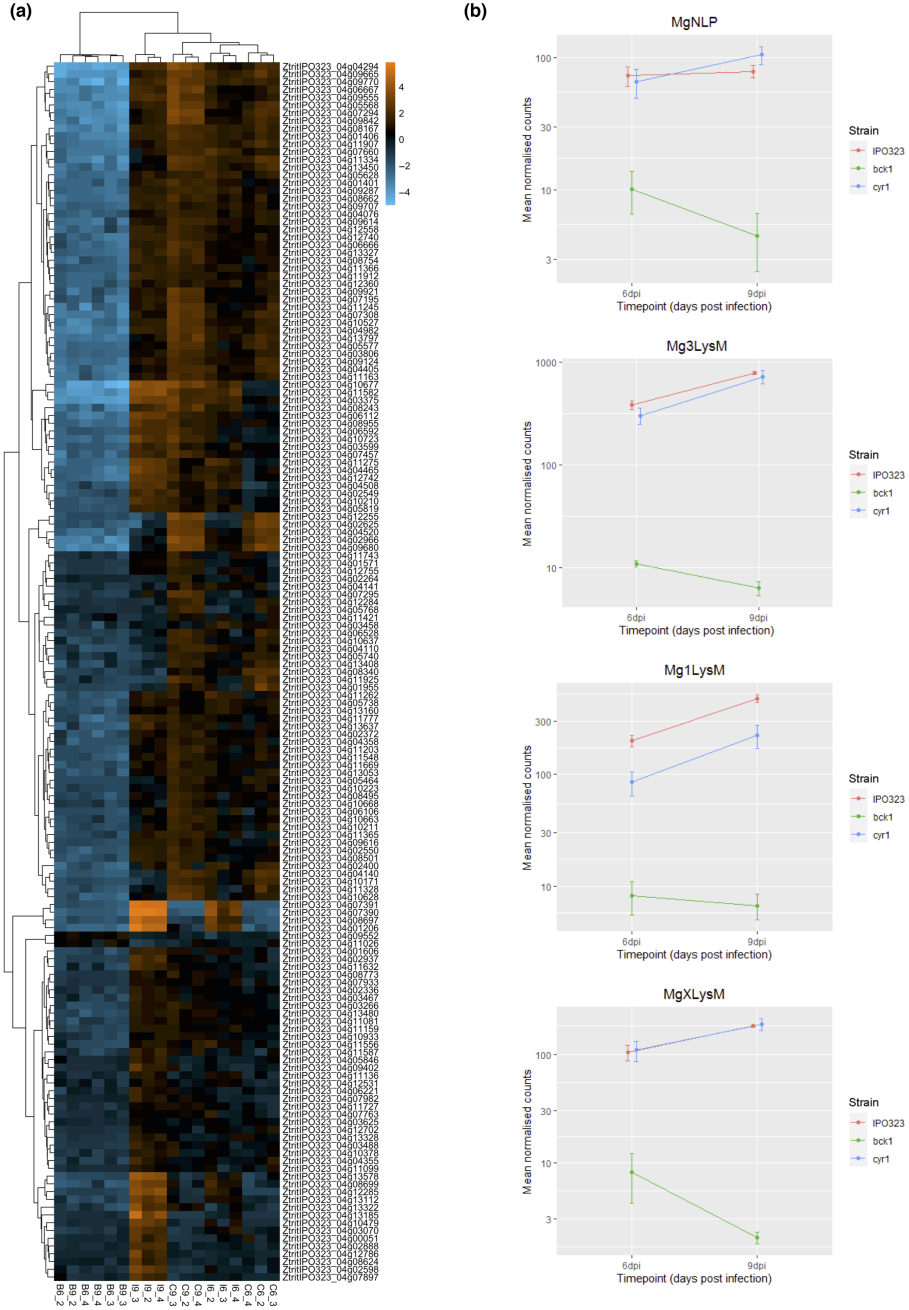
Putative secreted effector genes show differential regulation in Δ*ztbck1* and Δ*ztcyr1*. (a) Heat map displaying log‐fold change in normalized expression values relative to the mean normalized expression for differentially expressed effectors in each sample. Column labels (e.g., B6_2) indicate the sample strain (B, Δ*ztbck1*; C, Δ*ztcyr1*; I, IPO323), time point (6 and 9 days postinoculation [dpi]), and replicate (2, 3, and 4). (b) Mean of the normalized count values for *MgNLP* (ZtritIPO323_04g04358), *Mg3LysM* (ZtritIPO323_04g03143), Mg1LysM (ZtritIPO323_04g12742), and *MgXLysM* (ZtritIPO323_04g12740) in each strain at 6 and 9 dpi. Error bars represent standard error.

Furthermore, Δ*ztbck1* displayed widespread down‐regulation of genes encoding effector proteins (Figures 4a and 6a), predicted from the secretome using EffectorP v. 2.0 (Sperschneider et al., 2018). This includes the necrosis‐ and ethylene‐inducing protein 1 (Nep1)‐like protein MgNLP (Figure 6b), which is known to be highly up‐regulated during the late symptomless phase but is dispensable for virulence (Motteram et al., 2009). Crucially, all three LysM‐domain containing effectors, which are known to be *Z. tritici* virulence factors (Tian et al., 2021), are strongly down‐regulated in Δ*ztbck1* at both 6 and 9 dpi (Figure 6b). This finding suggests that host colonization by Δ*ztbck1* is inhibited by the inability of this strain to suppress the host defence response.

The sets of up‐ and down‐regulated genes in Δ*ztcyr1* compared to IPO323 from both time points were also significantly enriched with secretome genes (Figure 4a), the majority of which were functionally uncharacterized. All sets of Δ*ztcyr1* DEGs were significantly enriched for predicted effectors, the number of which increased between 6 and 9 dpi (Figure 4a). However, compared to their widespread down‐regulation in Δ*ztbck1* strains, the number of candidate effector DEGs was relatively few, indicating regulation of a more specific infection‐related gene set by the cAMP‐PKA pathway. Nineteen of the 27 effectors down‐regulated in Δ*ztcyr1* at 9 dpi were up‐regulated between 6 and 9 dpi in IPO323 (Figures 6a and S6a, and Table S3), suggesting that they function in the onset of necrotrophy. To support this, it was found that all of the effectors in this set of 27 displayed peak expression at the transition to, or during, the necrotrophic phase in a previous study (Rudd et al., 2015). As well as protein effectors, two polyketide synthase (PKS) genes and the hybrid PKS ribosomal peptide synthase *HSP1* (Rudd et al., 2015), which could function in biosynthesis of secondary metabolite effectors, were down‐regulated in Δ*ztcyr1* at 9 dpi (Figure S6b). These results indicate that the expression of effectors is diverging between Δ*ztcyr1* and the wild type as infection moves towards the necrotrophic phase.

Another striking difference in expression between Δ*ztcyr1* and IPO323 during infection was the up‐regulation of secreted proteases in the mutant (Figure 5b), which increased from 5 to 16 between 6 and 9 dpi (Table S3). All of these secreted proteases were found previously to show peak expression at the transition to necrotrophy during IPO323 infection, which was proposed to represent a switch to the use of host proteins as a carbon source (Rudd et al., 2015). Also heavily represented in the DEGs in Δ*ztcyr1* were the major facilitator superfamily (MFS) transporters, 18 and six of which were down‐ and up‐regulated, respectively, at 9 dpi in this strain (Table S2). Of these, 13 were annotated as sugar transporters (Figure S6c). Also down‐regulated at 9 dpi were three predicted amino acid transporters (Table S3). Together, these results suggest that genes potentially involved in acquisition of nutrients from the host are differentially regulated in Δ*ztcyr1* as infection progresses.

As well as showing differential expression of infection‐related secreted proteins, genes involved in cell wall biosynthesis were also found to be differentially regulated in Δ*ztbck1* during infection (Figure S7). A *Z. tritici* homologue of the *Aspergillus fumigatus* β‐1‐3‐glucanosyltransferase *ZtGel2*, involved in the formation of β‐1,3‐glucan branches, and an α‐1,3‐glucan synthase were both down‐regulated in Δ*ztbck1* compared to IPO323 at both time points (Figure S7a). Along with the sensitivity of Δ*ztbck1* to caspofungin and glucanase enzymes, this suggests that the *Z. tritici* CWI pathway is responsible for responding to β‐1,3‐glucan perturbation. Conversely, four chitin synthase genes were found to show significantly higher expression in Δ*ztbck1* during infection (Figure S7b). Enhanced expression of chitin synthases in Δ*ztbck1* could occur in response to cell wall damage caused by the unsuppressed host defence response, regulated by signalling mechanisms other than the CWI pathway.

### 2.8 Transcriptome profiling of wheat reveals different defence response to infection by Δ*ztcyr1* and Δ*ztbck1*

**FIGURE 7:**
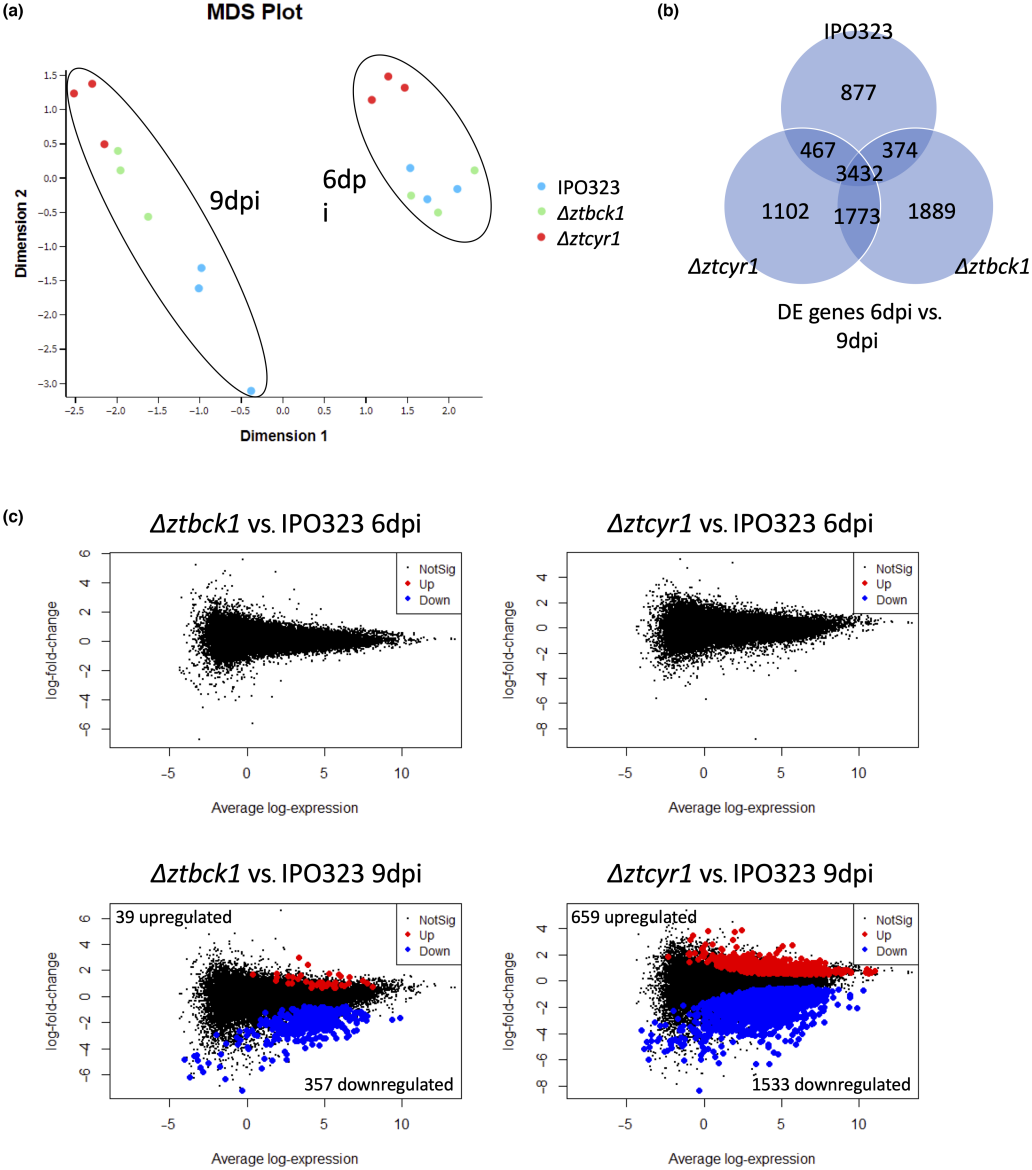
Global wheat gene expression analysis during infection. (a) Multidimensional scaling (MDS) plot on global wheat gene expression data. (b) Comparison of genes differentially expressed between 6 and 9 days postinoculation (dpi) in leaves infected with IPO323, Δ*ztbck1*, and Δ*ztcyr1*. (c) MA plots displaying differentially expressed genes between leaves infected with Δ*ztbck1* at 6 dpi (top left) and 9 dpi (bottom left), and Δ*ztcyr1* at 6 dpi (top right) and 9 dpi (bottom right), using leaves infected with IPO323 each time point as a reference.

Analysis of RNA sequencing reads aligned to the wheat genome was used to investigate whether the host response to Δ*ztcyr1* and Δ*ztbck1* infection was altered compared to colonization by the wild type. Clustering of samples based on global wheat gene expression data was investigated by multidimensional scaling (MDS) analysis (Figure 7a). This revealed that 40% of the variance between the samples can be summarized by the first dimension, which broadly segregates the samples by time point during infection at which they were taken (Figure 7a). When comparing wheat infected with each strain, a total of 3432 genes were differentially regulated between 6 and 9 dpi in the same direction (Figure 7b). *TaMPK3*, which is known to be involved in responses to various abiotic stresses in wheat (Goyal et al., 2018; Zhan et al., 2017), was significantly down‐regulated between 6 and 9 dpi in leaves infected with all the strains (Figure S8a). Furthermore, many RLK genes showed significant down‐regulation between 6 and 9 dpi in all infection experiments (Figure S8b and Table S4), expression of which is also known to respond to diverse abiotic stresses (Lehti‐Shiu et al., 2009; Shumayla, Kumar, et al., 2016; Shumayla, Pandey, et al., 2016). These findings suggest that all plants were under environmental stress at 6 dpi, which could have caused the large proportion of shared variation in global wheat gene expression observed between the time points in all experiments.

Despite this, samples did cluster depending on the inoculated *Z. tritici* strain; leaf samples infected with IPO323 and Δ*ztbck1* clustered separately from Δ*ztcyr1* at 6 dpi, before samples infected by IPO323 diverged further from Δ*ztcyr1* and Δ*ztbck1* at 9 dpi (Figure 7b). Despite the apparent difference in global transcription between samples infected by Δ*ztcyr1* and IPO323 at 6 dpi from MDS analysis, no statistically significant DEGs were identified between any strains at this time point (Figure 7c). However, a set of 396 DEGs were identified between Δ*ztbck1*‐ and IPO323‐infected samples at 9 dpi (Figure 7c and Table S4). Furthermore, 2192 DEGs were identified between Δ*ztcyr1*‐ and IPO323‐infected samples at 9 dpi (Figure 7c and Table S4).

**FIGURE 8:**
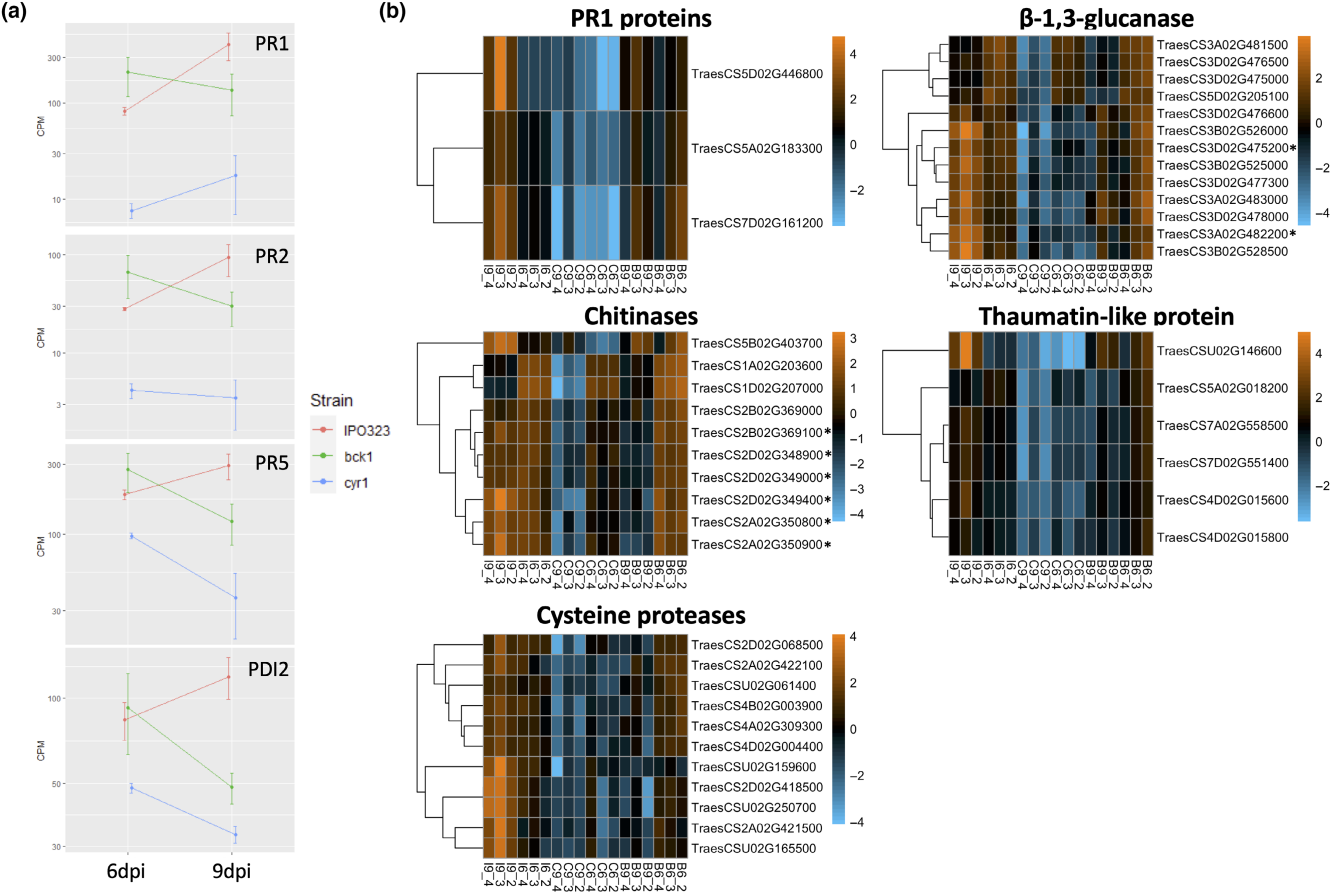
Defence‐related wheat genes show differential regulation in Δ*ztbck1*‐ and Δ*ztcyr1*‐infected leaves. (a) The mean counts per million (CPM) of each gene across samples infected with each strain at 6 and 9 days postinoculation (dpi) is plotted on a log scale, with error bars representing the standard error. (b) Heat maps displaying log‐CPM values relative to the mean of each row, scaled so that the standard deviation is 1 (*z*‐score). Plots display genes of defence‐related families that were differentially expressed in Δ*ztcyr1‐*infected samples at 9 dpi. Asterisks indicate differential expression in Δ*ztbck1*‐infected samples at 9 dpi. Column labels (e.g., B6_2) indicate the sample strain (B, Δ*ztbck1*; C, Δ*ztcyr1*; I, IPO323) and time point (6 and 9 dpi).

Further analysis of these DEG sets focused on wheat genes with predicted functions in defence responses against pathogens. Four genes characterized as responding to *Z. tritici* infection in a previous study (Ray et al., 2003) were significantly down‐regulated in Δ*ztcyr1* at 9 dpi (Figure 8a). This included the pathogenesis‐related (PR) proteins the PR1 protein *PR‐1‐4* (TraesCS7D02G161200), the β‐1,3‐glucanase *PR2* (TraesCS7D02G551400), and the thaumatin‐like protein *PR5* (TraesCS7D02G551400), as well as the protein disulphide isomerase *PDI2* (TraesCS4B02G101800). In addition to these genes, two other PR1 proteins, 12 other β‐1,3‐glucanases (PR2), 10 chitinases (PR3), and five other thaumatin‐like proteins (PR5) were found to be down‐regulated in Δ*ztcyr1*‐infected samples at 9 dpi (Figure 8b). A subset of β‐1,3‐glucanases and chitinases was also down‐regulated in Δ*ztbck1*‐infected samples at 9 dpi (Figure 8b).

Other protein annotations that were highly represented in the down‐regulated genes in Δ*ztcyr1* were proteases, including Cys‐ (Figure 8b), metallo‐ and subtilisin‐like families, ubiquitination proteins, and drug resistance ABC transporters (Table S3). Also highly represented in the DEGs within Δ*ztcyr1*‐infected leaves at 9 dpi were receptor‐like kinases (RLKs), including many from the wall‐associated, leucine‐rich repeat, cysteine‐rich, and lectin kinase families (Table S4). These protease and RLK families were previously characterized as transcriptionally associated with the transition to necrotrophy (Rudd et al., 2015). Furthermore, three LysM domain GPI‐anchored proteins of the chitin elicitor‐binding protein (CEBiP), which have been characterized in chitin‐induced defence responses to *Z. tritici* (Lee et al., 2014), were also down‐regulated at 9 dpi in Δ*ztcyr1* (Table S4). *TaMPK3*, a plant stress‐responsive mitogen activated protein kinase, which is induced by *Z. tritici* at the switch to necrotrophy (Rudd et al., 2008), was also significantly down‐regulated in Δ*ztcyr1* compared to IPO323‐infected leaves at 9 dpi. These findings strongly suggest that Δ*ztcyr1* is unable to induce the wheat defence response seen during wild‐type infection, despite continuing to grow in the host environment.

Regarding the host transcriptional response to Δ*ztbck1*, many of the aforementioned defence‐related genes were found amongst those down‐regulated between 6 and 9 dpi specifically in Δ*ztbck1* samples, and not in IPO323‐ or Δ*ztcyr1*‐infected leaves (Table S4). This suggests that the host defence response was induced in Δ*ztbck1*‐infected leaves at 6 dpi and subsequently declined at 9 dpi. This is supported by the fact that many of these defence‐related genes then become significantly down‐regulated in Δ*ztbck1*‐infected leaves compared to IPO323‐infected samples at 9 dpi (Figure 8b and Table S4), as the wild type progresses towards the necrotrophic phase and induction of the hypersensitive host response. Along with the finding that Δ*ztbck1* fungal biomass does not increase between the time points, this suggests that Δ*ztbck1* colonization is inhibited early on by the host defence response, which starts to subside as less fungal penetration events occur.

## 3 DISCUSSION

The hyphal growth rate of Δ*ztbck1* was found to be reduced during prolonged growth on WA, similar to the previously characterized Δ*mgslt2* mutant (Mehrabi et al., 2006). This indicates that disruption of the CWI pathway may have a negative impact on *Z. tritici* polarized growth through deregulation of proteins involved in cell wall biosynthesis at the hyphal apex. However, despite causing defects in vegetative growth in vitro, deletion of *ZtBCK1* did not influence the ability of *Z. tritici* spores to germinate on the leaf surface and grow to the stomatal aperture. Furthermore, no evidence was found for the growth of Δ*ztbck1* mutants beyond the symptomless phase, with no increase in fungal biomass detected after 6 dpi. These findings are consistent with the phenotypes identified in Δ*mgslt2* mutants, which were unable to colonize the mesophyll after entering the substomatal cavities (Mehrabi et al., 2006), and point towards the role of the CWI pathway in regulating adaptation to the host environment.

Transcriptome profiling revealed that Δ*ztbck1* was severely inhibited in the expression of predicted secreted proteins during infection. This included the three *Z. tritici* LysM effectors, which are known to have partially redundant functions in the evasion and tolerance of the chitin‐induced wheat defence response, and together are essential for full *Z. tritici* virulence (Marshall et al., 2011; Tian et al., 2021). Furthermore, wheat expression analysis revealed the high expression of numerous defence‐related genes in response to infection by Δ*ztbck1*, including many bona fide PR proteins. Together, these findings suggest that the CWI pathway is involved in regulation of virulence‐associated secreted proteins in response to the host apoplast, and that this is required for suppression of the host immune response to enable mesophyll colonization.

In *S. cerevisiae*, the CWI pathway is required for controlling cell wall homeostasis in response to stress caused by hydrolytic enzymes and cell wall‐perturbing agents, as well as heat, osmotic, and pH stress (Garcia et al., 2009; Kamada et al., 1995; Reinoso‐Martín et al., 2003). Here, Δ*ztbck1* mutants were found to have increased sensitivity to β‐1,3‐glucanase enzymes and the β‐1,3‐glucan synthesis‐inhibiting echinocandin fungicide caspofungin, but not to chitin disruption by calcofluor white. This corroborates previous findings that deletion of *MgSLT2* increases sensitivity to glucanase but not chitinase enzymes (Mehrabi et al., 2006). Furthermore, genes involved in biosynthesis of β‐1,3‐glucan and α‐1,3‐glucan were found to be down‐regulated in Δ*ztbck1* during infection, while chitin synthases were up‐regulated in this strain. Increased chitin synthase expression in Δ*ztbck1* could occur in response to the cell wall perturbation caused by the enhanced host defence response to this strain, without the protective influence of LysM effectors against host chitinases (Marshall et al., 2011). These findings suggest that the *Z. tritici* CWI signalling specifically regulates responses to glucan perturbation, while responses to chitin disruption are regulated by a separate pathway, such as the HOG1 MAPK and calcineurin pathways (Bruder Nascimento et al., 2016; Fortwendel et al., 2010; Munro et al., 2007).

Considering this, we hypothesize that the CWI pathway in *Z. tritici* has adapted to co‐regulate secreted proteins involved in cell wall remodelling and virulence‐related functions, explaining the down‐regulation of these genes in Δ*ztbck1* during infection. These findings raise the possibility that *ZtBCK1* regulates secreted protein expression during infection following cell wall perturbation by host β‐1,3‐glucanases in the apoplast. Alternatively, this could occur on recognition of other environmental stimuli in the apoplast, such as the acidic pH, response to which is known to be controlled by the CWI pathway in yeast (Claret et al., 2005).

Unlike Δ*ztbck1* mutants, Δ*ztcyr1* strains continued to grow within the host late into the symptomless phase. This is consistent with the phenotypes of deletion mutants in the catalytic and regulatory PKA subunit genes, which were able to extensively colonize the mesophyll tissue (Mehrabi & Kema, 2006). This suggests that the function of *ZtCYR1* in *Z. tritici* infection is distinct from the role of cAMP‐PKA signalling in host penetration by appressorium‐forming *M. oryzae* and *Colletotrichum* sp. (Choi & Dean, 1997; Yamauchi et al., 2004), and even fungal phytopathogens that do not develop true appressoria such as *F. graminearum* (Bormann et al., 2014).

While the Δ*ztcyr1* mutants have completely abolished virulence, previously characterized deletion strains in the *Z. tritici* PKA subunit *MgTPK2* are still able to cause delayed necrosis, but do not develop pycnidia (Mehrabi & Kema, 2006). Furthermore, the sensitivity to high osmolarity and defective melanization of Δ*ztcyr1* is more similar to those previously observed in *Z. tritici* mutants lacking the PKA regulatory subunit (MgBCY1) than the catalytic subunit MgTPK2 (Mehrabi & Kema, 2006). This suggests that *MgTPK2* has redundant function with the second PKA catalytic subunit gene in *Z. tritici MgTPK1* (ZtritIPO323_04g08063), as in other ascomycete pathogens (Fuller et al., 2011; Hu et al., 2014; Li et al., 2017). Similar phenotypes of regulatory PKA subunit and adenylate cyclase mutants may be surprising, such as those observed here and in *M. oryzae* (Selvaraj et al., 2017), as BCY1 inhibits the activity of PKA catalytic subunits, while CYR1 activates this pathway. However, BCY1 is known to control the nuclear localization of PKA in *S. cerevisiae* and *Candida albicans* (Cassola et al., 2004; Griffioen et al., 2001). Combined, these results suggest that *MgBCY1* has additional roles in regulating the PKA pathway beyond its inhibitory effect, which are required for correct PKA function.

The Δ*ztcyr1* strains were also found to display increased sensitivity to caspofungin and osmotic stress. Although PKA signalling is inhibited during cell wall stress in *S. cerevisiae* (García et al., 2017, 2019), positive regulation of the cell wall stress response through crosstalk between the CWI and PKA pathways was identified in *Cryptococcus neoformans* (Donlin et al., 2014). Furthermore, PKA has been shown to facilitate cell wall remodelling in response to cell wall and osmotic stress in *A. fumigatus* through its involvement in carbohydrate mobilization in conjunction with the high osmolarity glycerol (HOG) pathway (de Assis et al., 2018; Shwab et al., 2017). This provides a potential mechanism by which PKA signalling may contribute to the response to cell wall and osmotic stress in *Z. tritici*.

Along with the previously characterized role of PKA in *Z. tritici* asexual development (Mehrabi & Kema, 2006), the results presented here suggest that cAMP signalling is required for the induction of necrosis during infection. A similar function of the cAMP‐PKA pathway in controlling invasive growth has been identified in *M. oryzae*, in which Δ*cpka*Δ*cpk1* mutants were unable to cause necrosis when injected into the rice leaf (Li et al., 2017). Transcriptome analysis found that gene expression in Δ*ztcyr1* was similar to IPO323 during the symptomless phase at 6 dpi but diverged as infection moved towards the transition to necrotrophy. This divergence in expression was particularly noticeable in predicted effector genes, including many which display peak expression around the necrotrophic switch (Rudd et al., 2015). Furthermore, analysis of wheat gene expression revealed that Δ*ztcyr1*‐infected leaves show widespread down‐regulation of defence‐related genes compared to wild‐type‐infected leaves at 9 dpi. This strongly suggests that Δ*ztcyr1* is unable to induce the immune response required for the onset of necrosis. Those effector genes that are strongly down‐regulated in Δ*ztcyr1* at 9 dpi may therefore be involved in inducing the host hypersensitive response, warranting further functional characterization.

In conclusion, this study provides evidence that the CWI pathway regulates the transcriptional response to the host environment in *Z. tritici*, which goes beyond genes involved in cell wall remodelling to activate expression of effector proteins. This includes virulence factors known to be required for suppression of the chitin‐triggered host immune response. This points towards the possible co‐regulation of the cell wall stress response with virulence‐related genes in response to the host mesophyll environmental. Furthermore, this study implicates the PKA pathway in controlling the switch to necrotrophic growth in addition to its previously characterized function in asexual development. The absence of *ZtCYR1* influences the expression of putative effectors and genes involved in accessing nutrients around the transition to necrotrophy, which may disrupt the induction of host necrosis. These findings further our understanding of how CWI and cAMP signalling contribute to *Z. tritici* pathogenicity. Further study is required of the signals that activate both pathways during infection, as well as the regulatory components that detect these signals.

## 4 EXPERIMENTAL PROCEDURES

### 4.1 Strains and growth conditions

The wild type *Z. tritici* strain IPO323 was used in this study. Yeast‐like *Z. tritici* cells were stored long‐term in suspensions of 50% glycerol at −80°C and cultured on yeast extract peptone dextrose (YPD; 1% yeast extract, 2% peptone, 2% glucose) agar at 19°C under darkness for 5 days before use in transformations, infection experiments, and in vitro phenotypic assays.

### 4.2 Whole‐genome resequencing

To extract DNA, 100 mg of *Z. tritici* yeast‐like cells was collected from YPD agar cultures, snap frozen in liquid nitrogen, and ground in a pestle and mortar. DNA was extracted using the illustra Nucleon Phytopure Genomic DNA Extraction Kit (GE Healthcare) following the manufacturer's protocol, including the RNAse A digestion step. Extracted DNA was then further purified using the DNeasy Plant Mini Kit (Qiagen). Whole‐genome resequencing was carried out by BGI Tech Solutions Co., Ltd (Hong Kong) using their BGISEQ‐500 platform for paired‐end 150 bp reads. Bioinformatic analysis is described in detail in File S1. In brief, reads were trimmed using fastp (Chen et al., 2018) and aligned with the IPO323 genome with Bowtie 2 (Langmead & Salzberg, 2012), before variant calling using FreeBayes (Garrison & Marth, 2012).

### 4.3 Construction of plasmid vectors

The plasmids pC‐HYG‐CYR1KO and pC‐HYG‐BCK1KO were constructed by homologous recombination in yeast using the plasmid pC‐HYG‐YR following the previously described method (Sidhu et al., 2015). PCR amplification of left flank (LF) and right flank (RF) regions from either side of the *ZtBCK1* and *ZtCYR1* coding sequences was carried out using the primer pairs ZtCYR1‐LF‐F/R, ZtCYR1‐RF‐F/R, ZtBCK1‐LF‐F/R and ZtBCK1‐RF‐F/R (Table S1). LF and RF amplicons were transformed into *S. cerevisiae* alongside the pC‐HYG‐YR vector, which had been linearized by restriction digestion with the enzymes EcoRI and HindIII (New England Biolabs). The sequences of flanking regions were confirmed to be correct in the completed vectors by Sanger sequencing. These vectors were transformed into *Z. tritici* via *Agrobacterium tumefaciens*‐mediated transformation following the protocol deposited on protocols.io (Child & Helmstetter, 2022), which describes modifications to the original method (Zwiers & de Waard, 2001).

### 4.4 Enzymatic spore lysis assays

The ability of *Z. tritici* strains to withstand disruption of cell wall integrity was assessed using lyticase from *Arthrobacter luteus* (Sigma‐Aldrich). Cells were harvested from YPD agar cultures and suspended at a concentration of 5 × 10^6^ spores/mL in sterile water containing a range of concentrations of lyticase enzyme, following a two‐fold dilution from 40 to 0.625 U/mL. Suspensions were incubated at 25°C for 2 h on an orbital shaker (120 rpm) before cell suspensions were diluted 40 fold and 100 μL plated onto YPD agar plates. The viability of *Z. tritici* after lyticase treatment was assessed by observing growth after 5 days at 19°C. The experiment was repeated three times.

### 4.5 Wheat infection assays

Wheat leaves (cultivar Riband) were infected with *Z. tritici* strains IPO323, Δ*ztcyr1.4* and Δ*ztbck1.4* at a concentration of 5 × 10^6^ spores/mL following the previously described method (Motteram et al., 2009). Leaf samples for RNA extractions were harvested at 6 and 9 dpi, with each technical replicate being generated from six independent seedling (biological replicate) leaf sections (c.6 cm each) infected with the same *Z. tritici* strain, and frozen immediately in liquid nitrogen before storage at −80°C. Three technical replicates of each experiment were carried out.

### 4.6 RNA sequencing

RNA was extracted from *Z. tritici*‐infected wheat leaf tissue using TRIzol reagent (Fisher Scientific). Samples were ground in liquid nitrogen using a pestle and mortar and RNA was extracted with TRIzol reagent following the manufacturer's protocol apart from phase separation with 1‐bromo‐3‐chloropropane. RNA was purified using the RNeasy Plant Mini Kit (Qiagen), following the RNA Cleanup protocol including an on‐column DNA digestion step using the RNase‐free DNase set (Qiagen).

Libraries were prepared using the TruSeq HT stranded mRNA preparation kit following the manufacturer's protocol and sequenced as paired‐ends using an Illumina Novaseq 6000 on an S4 flow cell at 2 × 150 bp.

### 4.7 Alignments and differential expression analysis

Details of RNA sequencing analysis are given in File S1. In brief, reads were adapter‐ and quality‐trimmed with fastp before alignment to the *Z. tritici* genome (indexed with gene annotations from Rothamsted Research; King et al., 2017) and calculation of gene counts was carried out with STAR (Dobin et al., 2013). Normalization of gene counts and differential expression analysis was carried out using DESeq2 (Love et al., 2014), identifying DEGs between each mutant strain and the wild type at each time point, and between 6 and 9 dpi for each strain. Wald test *p* values were adjusted for multiple testing using the Benjamin–Hochberg (BH) correction method, and genes were deemed as differentially expressed when *p*_adj_ <0.01.

RSEM (with alignment using STAR) was chosen for wheat transcript quantification as it factors in the high number of multimapped reads, which result from the polyploidy of the wheat genome, using an expectation maximization (EM) algorithm (Deschamps‐Francoeur et al., 2020; Li & Dewey, 2011). Expected gene‐level counts were filtered and normalized using the trimmed mean of M‐values (TMM) method in EdgeR (Robinson et al., 2010; Robinson & Oshlack, 2010), before the limma package was used for differential expression analysis (Ritchie et al., 2015). Genes were identified as differentially expressed between treatments using the moderated *t* test within the *topTable* function, testing whether the log_2_‐fold‐change values for a particular contrast differ from 0 at a BH‐corrected *p*_adj_ threshold of 0.01.

## Supporting information


**FIGURE S1** Identification of avirulent *Zymoseptoria tritici* T‐DNA insertion strains

**FIGURE S2** Strains C5 and L2 contain T‐DNA insertions at targeted loci and a nonsynonymous point mutation in the *ZtCYR1* gene

**FIGURE S3** Identification of T‐DNA insertion upstream of, and deletion within, *ZtBCK1* in the avirulent *Zymoseptoria tritici* strain T21

**FIGURE S4** Diagrams showing strategy for deletion of *ZtCYR1* and *ZtBCK1*

**FIGURE S5** In vitro phenotypes of Δ*ztbck1* and Δ*ztcyr1* under cell wall and osmotic stress

**FIGURE S6** Differentially expressed effector, polyketide synthase, and major facilitator superfamily genes in Δ*ztcyr1*

**FIGURE S7** Cell wall biosynthesis enzymes are differentially expressed in Δ*ztbck1*

**FIGURE S8** Receptor‐like kinases and TaMPK3 expression is down‐regulated in Δ*ztbck1* and Δ*ztcyr1* at 9 days postinoculation

**TABLE S1** Primers used in this study

**TABLE S2** Polymorphisms identified in avirulent T‐DNA insertion strains

**TABLE S3** Differentially expressed *Zymoseptoria tritici* genes during infection by Δ*ztbck1* and Δ*ztcyr1* compared to IPO323 at 6 and 9 days postinoculation (dpi), and between the time points for each strain

**TABLE S4** Differentially expressed wheat genes during infection by Δ*ztbck1* and Δ*ztcyr1* compared to IPO323 at 9 days postinoculation (dpi), and between 6 and 9 dpi for each strain

**FILE S1** Supplementary methods. Details of bioinformatics methods used in this study

## Data Availability

RNA sequencing reads have been deposited on the European Nucleotide Archive (ENA) at www.ebi.ac.uk/ena under the accession number PRJEB58154.
