# Supplementary material for: Comparison of the impact of two key fungal signalling pathways on Zymoseptoria tritici infection reveals divergent contribution to invasive growth through distinct regulation of infection‐associated genes
Source: Mol Plant Pathol. 2023 Jun 12;24(10):1220–37. doi: 10.1111/mpp.13365 (PMC10502814; doi:10.1111/mpp.13365)
Supplement: Supplementary file 1 — FIGURE S1 Identification of avirulent Zymoseptoria tritici T‐DNA insertion strains [file MPP-24-1220-s008.docx]

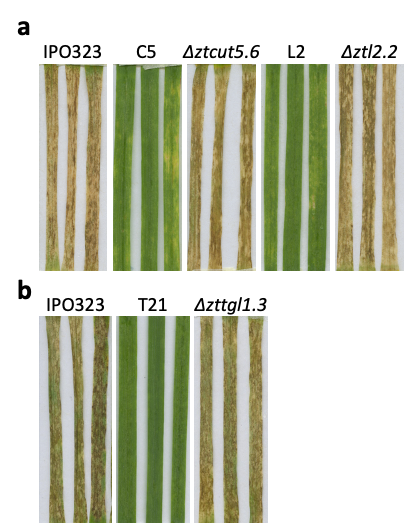


**Figure S1. Identification of avirulent *Z. tritici* T-DNA insertion strains**

Disease symptoms on wheat leaves after 21 days infection by *Z. tritici* strains (a) C5 and L2 compared to wild type IPO323 and representative independent *Δztcut5* and *Δztl2* deletion strains, and (b) T21 compared to IPO323 and a confirmed *Δzttgl1* deletion strain.
