# Supplementary material for: Comparison of the impact of two key fungal signalling pathways on Zymoseptoria tritici infection reveals divergent contribution to invasive growth through distinct regulation of infection‐associated genes
Source: Mol Plant Pathol. 2023 Jun 12;24(10):1220–37. doi: 10.1111/mpp.13365 (PMC10502814; doi:10.1111/mpp.13365)
Supplement: Supplementary file 2 — FIGURE S2 Strains C5 and L2 contain T‐DNA insertions at targeted loci and a nonsynonymous point mutation in the ZtCYR1 gene [file MPP-24-1220-s003.docx]

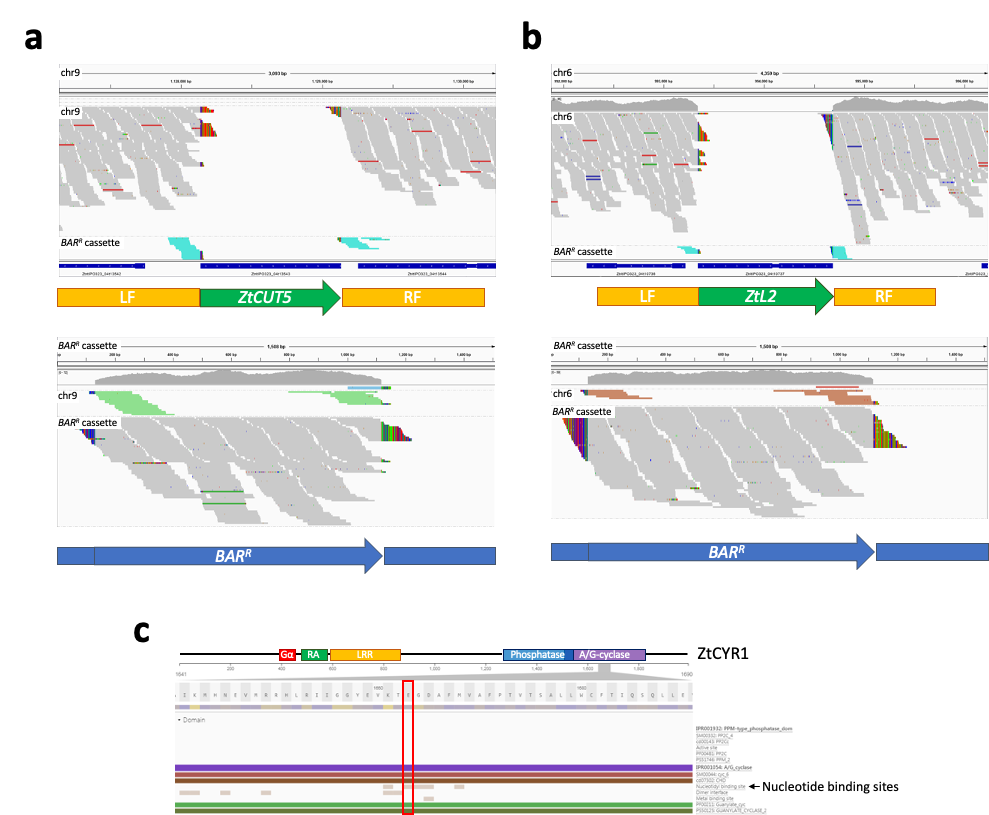


**Figure S2 Strains C5 and L2 contain T-DNA insertions at targeted loci and a nonsynonymous point mutation in the *ZtCYR1* gene.**

Integrative Genomics Viewer screenshots of strains (a) C5 and (b) L2 whole genome resequencing reads aligned at the *ZtCUT5* and *ZtL2* loci, respectively, in the *Z. tritici* IPO323 genome. Absence of reads aligned to coding sequences of genes and reads at the 3’ end of the left flank (LF) and 5’ end of the right flank (RF) aligning to the *BAR^R^* cassette. Reads are clustered vertically by the chromosome to which their pair is aligned. (c) Site of the point mutation (red box) in the A/G-cyclase domain of *ZtCYR1* identified in both C5 and L2, with the *ZtCYR1* domain structure (above) and the domains and features identified by InterProScan in the region of the mutation, including the putative nucleotide binding site at the mutation position.
