# Supplementary material for: Comparison of the impact of two key fungal signalling pathways on Zymoseptoria tritici infection reveals divergent contribution to invasive growth through distinct regulation of infection‐associated genes
Source: Mol Plant Pathol. 2023 Jun 12;24(10):1220–37. doi: 10.1111/mpp.13365 (PMC10502814; doi:10.1111/mpp.13365)
Supplement: Supplementary file 3 — FIGURE S3 Identification of T‐DNA insertion upstream of, and deletion within, ZtBCK1 in the avirulent Zymoseptoria tritici strain T21 [file MPP-24-1220-s006.docx]

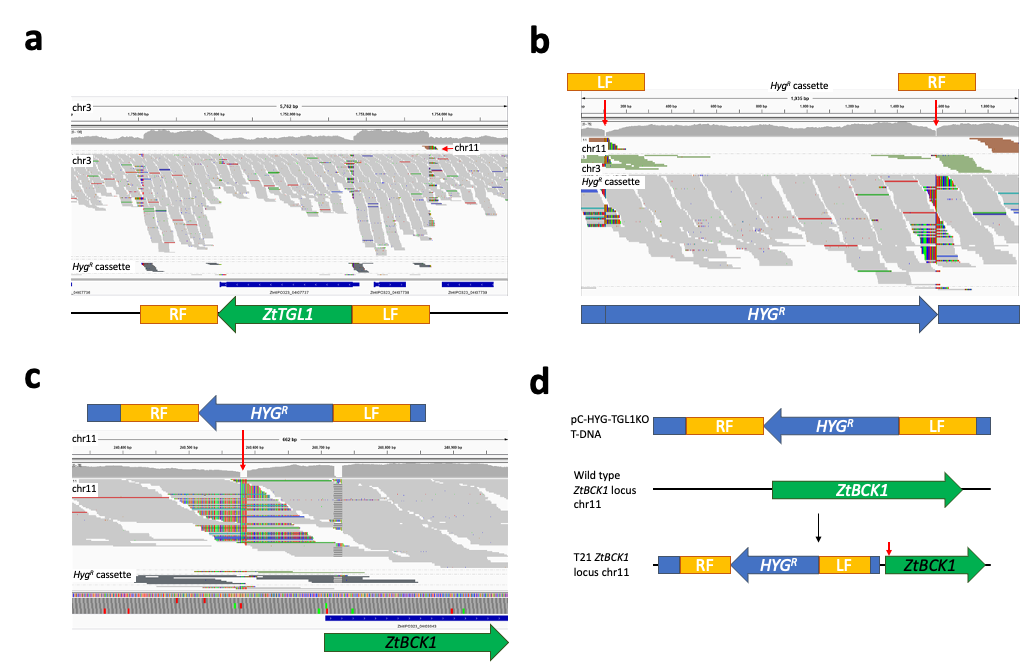


**Figure S3. Identification of T-DNA insertion upstream of, and deletion within, *ZtBCK1* in the avirulent *Z. tritici* strain T21.**

(a) T21 reads aligned to *ZtTGL1* locus showing presence of wild type allele and increased coverage over each flanking region (LF and RF), indicating the presence of these sequences elsewhere in the genome. Reads aligning to either end of the flanking sequences have pairs aligning to the *HYG^R^* cassette. (b) Reads aligned to the T-DNA sequence transformed into this strain, showing reads aligning to the 5’ and 3’ ends of the sequence whose pairs align to chromosome 11. Red arrows represent locations of LF and RF sequences in inserted T-DNA, which are surrounded by aligned reads whose pairs are mapped to the loci of LF and RF in chromosome 3. (c) Reads aligned to the *ZtBCK1* locus on chromosome 11. Red arrow represents site of T-DNA insertion, showing reads aligned either side of this site whose pairs are mapped to the T-DNA sequence. A deletion was identified at the start of the *ZtBCK1* coding sequence (d) Diagram displaying insertion of T-DNA upstream of *ZtBCK1* in strain T21 and the deletion site (red arrow) in the *ZtBCK1* coding sequence.
