# Supplementary material for: Comparison of the impact of two key fungal signalling pathways on Zymoseptoria tritici infection reveals divergent contribution to invasive growth through distinct regulation of infection‐associated genes
Source: Mol Plant Pathol. 2023 Jun 12;24(10):1220–37. doi: 10.1111/mpp.13365 (PMC10502814; doi:10.1111/mpp.13365)
Supplement: Supplementary file 4 — FIGURE S4 Diagrams showing strategy for deletion of ZtCYR1 and ZtBCK1 [file MPP-24-1220-s004.docx]

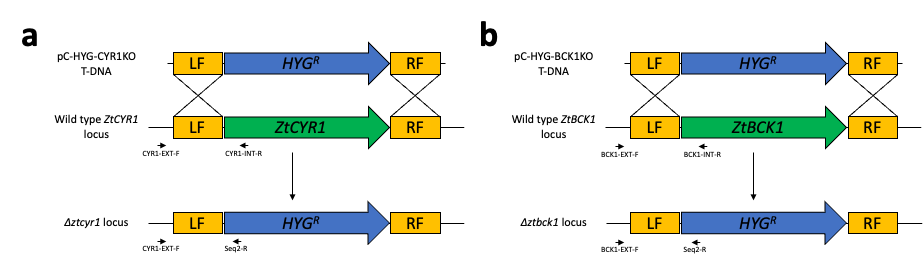


**Figure S4 Diagrams showing strategy for deletion of *ZtCYR1 and* *ZtBCK1.***

Strategy for deletion of (a) *ZtCYR1 and* (b) *ZtBCK1* using the plasmids pC-HYG-CYR1KO and pC-HYG-BCK1KO, respectively, with primers used to screen transformants under their respective target loci.
