# Supplementary material for: Comparison of the impact of two key fungal signalling pathways on Zymoseptoria tritici infection reveals divergent contribution to invasive growth through distinct regulation of infection‐associated genes
Source: Mol Plant Pathol. 2023 Jun 12;24(10):1220–37. doi: 10.1111/mpp.13365 (PMC10502814; doi:10.1111/mpp.13365)
Supplement: Supplementary file 5 — FIGURE S5 In vitro phenotypes of Δztbck1 and Δztcyr1 under cell wall and osmotic stress [file MPP-24-1220-s009.docx]

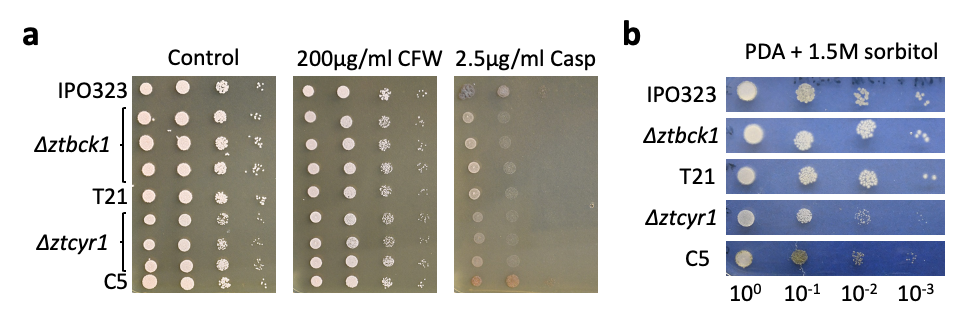


**Figure S5. *In vitro* phenotypes of *Δztbck1* and *Δztcyr1* under cell wall and osmotic stress.** (a) Sensitivity assays of *Z. tritici* strains grown on YPD supplemented with calcofluor white (CFW) and caspofungin (Casp). 5 μl droplets of a 10-fold serial dilution starting at a concentration of 5x10^6^ spores/ml. (b) *Z. tritici* grown for 10 days on PDA containing sorbitol at 19^o^C, 5 μl droplets of a 10-fold serial dilution starting at a concentration of 5x10^6^ spores/ml.
