# Supplementary material for: Comparison of the impact of two key fungal signalling pathways on Zymoseptoria tritici infection reveals divergent contribution to invasive growth through distinct regulation of infection‐associated genes
Source: Mol Plant Pathol. 2023 Jun 12;24(10):1220–37. doi: 10.1111/mpp.13365 (PMC10502814; doi:10.1111/mpp.13365)
Supplement: Supplementary file 6 — FIGURE S6 Differentially expressed effector, polyketide synthase, and major facilitator superfamily genes in Δztcyr1 [file MPP-24-1220-s002.docx]

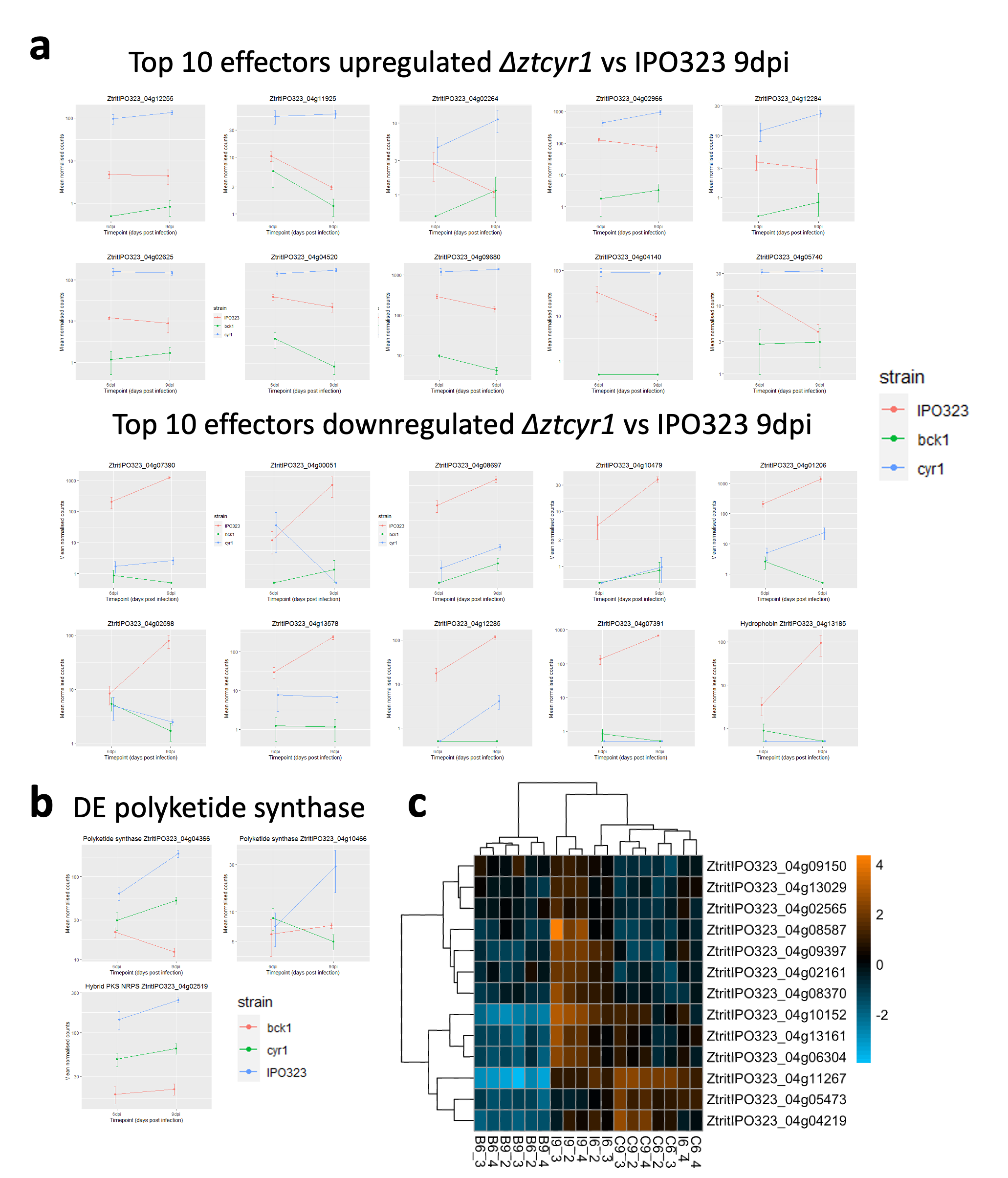


**Figure S6. Differentially expressed effector, polyketide synthase and major facilitator superfamily genes in *Δztcyr1.***

Expression profiles of putative effectors that were upregulated and downregulated by the largest fold-change (a), as well as differentially expressed polyketide synthase genes (b), in *Δztcyr1* at 9 dpi compared to IPO323. Mean of the normalised count values in each strain at 6 dpi and 9 dpi are plotted, with error bars representing standard error. (c) Heat map displaying log-fold change in normalised expression values relative to the mean normalised expression for major facilitator superfamily (MFS) sugar transporters that were differentially expressed between *Δztcyr1* and IPO323 at 9 dpi. Column labels (e.g. B6_2) indicate the sample strain (B=*Δztbck1*, C=*Δztcyr1,* I=IPO323), time point (6 dpi and 9 dpi) and replicate (2, 3 and 4).
