# Supplementary material for: Comparison of the impact of two key fungal signalling pathways on Zymoseptoria tritici infection reveals divergent contribution to invasive growth through distinct regulation of infection‐associated genes
Source: Mol Plant Pathol. 2023 Jun 12;24(10):1220–37. doi: 10.1111/mpp.13365 (PMC10502814; doi:10.1111/mpp.13365)
Supplement: Supplementary file 7 — FIGURE S7 Cell wall biosynthesis enzymes are differentially expressed in Δztbck1 [file MPP-24-1220-s007.docx]

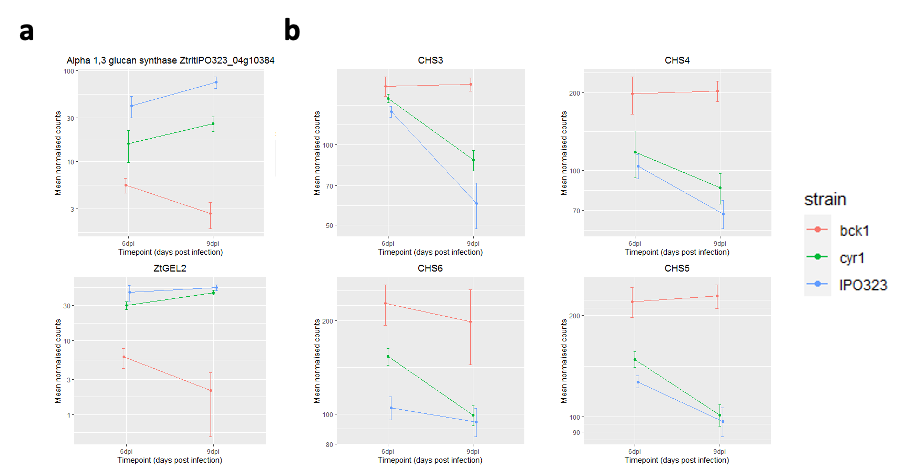


**Figure S7. Cell wall biosynthesis enzymes are differentially expressed in *Δztbck1*.**

Mean of the normalised count values in each strain at 6 dpi and 9 dpi for (A) alpha-1,3-glucan synthase (ZtritIPO323_04g10384) and *ZtGEL2* (ZtritIPO323_04g13416) and (B) differentially expressed chitin synthase genes (*CHS3*/ZtritIPO323_04g00292, *CHS4*/ZtritIPO323_04g09937, *CHS5/*ZtritIPO323_04g06329 and *CHS6*/ZtritIPO323_04g06328. Error bars represent standard error.
