# Supplementary material for: Comparison of the impact of two key fungal signalling pathways on Zymoseptoria tritici infection reveals divergent contribution to invasive growth through distinct regulation of infection‐associated genes
Source: Mol Plant Pathol. 2023 Jun 12;24(10):1220–37. doi: 10.1111/mpp.13365 (PMC10502814; doi:10.1111/mpp.13365)
Supplement: Supplementary file 8 — FIGURE S8 Receptor‐like kinases and TaMPK3 expression is down‐regulated in Δztbck1 and Δztcyr1 at 9 days postinoculation [file MPP-24-1220-s011.docx]

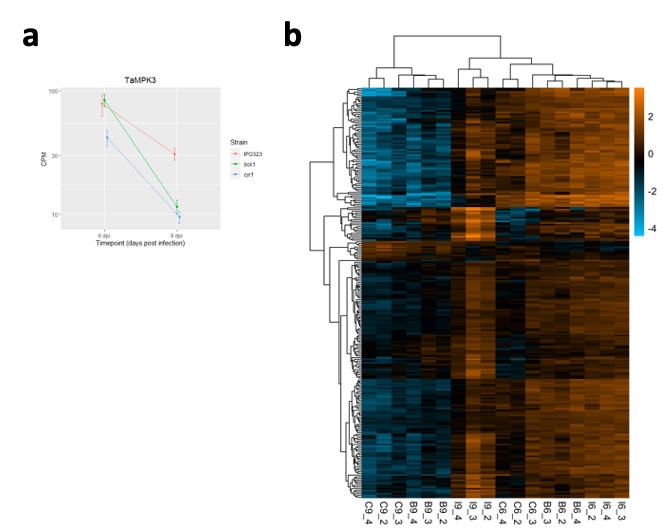


**Figure S8. Receptor-like kinases and TaMPK3 expression is downregulated in *Δztbck1* and *Δztcyr1* at 9dpi.**

(a) Expression profile of *TaMPK3* (TraesCS4A02G106400) across samples, displaying mean counts per million across repeated samples from same treatment and error bars displaying standard error. (b) Expression profiles of receptor-like kinases that were differentially expressed in *Δztbck1* and *Δztcyr1* infected leaves at 9dpi. Heat map displaying log-CPM values relative to the mean of each row, scaled so that the standard deviation is one (z-score). Column labels (e.g. B6_2) indicate the sample strain (B=*Δztbck1*, C=*Δztcyr1,* I=IPO323), time point (6 dpi and 9 dpi) and replicate (2, 3 and 4).
