# Supplementary material for: Comparison of the impact of two key fungal signalling pathways on Zymoseptoria tritici infection reveals divergent contribution to invasive growth through distinct regulation of infection‐associated genes
Source: Mol Plant Pathol. 2023 Jun 12;24(10):1220–37. doi: 10.1111/mpp.13365 (PMC10502814; doi:10.1111/mpp.13365)
Supplement: Supplementary file 9 — TABLE S1 Primers used in this study [file MPP-24-1220-s010.docx]

**Table S1. Primers used in this study**

| Primer name | Sequence (5’–3’) |
| --- | --- |
| ZtBCK1_del_seq_F | GCGCATACACATACGACCTC |
| ZtBCK1_del_seq_R | GCGCGACTGACTTTGTGATT |
| ZtBCK1_insert_seq_F | TAGCTTCGCCGCTACGTTTC |
| ZtBCK1_insert_seq_R | AGGTATGTAGCCTCCGACGG |
| ZtBCK1_LF_F | CTAGGCCACCATGTTGGGCCCGGCGCGCCGCGATGAAGATTGACACGCT |
| ZtBCK1_LF_R | TCCTTCAATATCAGTTGGGTACCGAGCTCGTTCGCTGAATCTGCCTTTC |
| ZtBCK1_RF_F | GATCCTCTAGAGTCGACCTGCAGGCATGCCGCAGCATAGGAAAGTGTATGG |
| ZtBCK1_RF_R | GTCAGATCTACCATGGTGGACTCCTCTTACTCCTTTGATGTCCGGGACT |
| ZtBCK1_EXT_F | TGGATTACCCGCAGAGCAAG |
| ZtBCK1_INT_R | TCGGAGCAGATTCCTGTCTC |
| ZtCYR1_SNP_seq_F | GAACAGCACGATCTTGTGGG |
| ZtCYR1_SNP_seq_R | CGTTGGACTTCCTGGATGAA |
| ZtCYR1_LF_F | CTAGGCCACCATGTTGGGCCCGGCGCGCCCCGAACAATCTCCCAGCTAC |
| ZtCYR1_LF_R | TCCTTCAATATCAGTTGGGTACCGAGCTCCCATCGAGGCTCAGCGGATT |
| ZtCYR1_RF_F | GATCCTCTAGAGTCGACCTGCAGGCATGCGCAGTCGTCGGATGCCTGAC |
| ZtCYR1_RF_R | GTCAGATCTACCATGGTGGACTCCTCTTACACACATCGCGAACACACAC |
| ZtCYR1_EXT_F | GGTGAATAGGCGTGCAGATG |
| ZtCYR1_INT_R | CTGAACCATCCTCCTGCGAA |
| Seq2-R | TTGTTGACCTCCACTAGCTC |
