# Supplementary figures and images for: Comparison of the impact of two key fungal signalling pathways on Zymoseptoria tritici infection reveals divergent contribution to invasive growth through distinct regulation of infection‐associated genes
Source: Mol Plant Pathol. 2023 Jun 12;24(10):1220–37. doi: 10.1111/mpp.13365 (PMC10502814; doi:10.1111/mpp.13365)

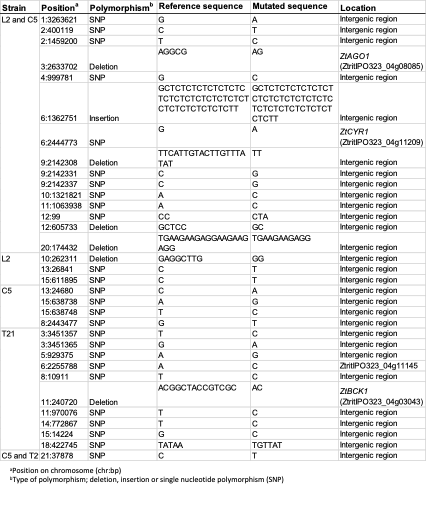


**Table S2. Polymorphisms identified in avirulent T-DNA insertion strains.**

Supplement: Supplementary file 10 — TABLE S2 Polymorphisms identified in avirulent T‐DNA insertion strains [file MPP-24-1220-s012.docx]
