# Supplementary material for: Comparison of the impact of two key fungal signalling pathways on Zymoseptoria tritici infection reveals divergent contribution to invasive growth through distinct regulation of infection‐associated genes
Source: Mol Plant Pathol. 2023 Jun 12;24(10):1220–37. doi: 10.1111/mpp.13365 (PMC10502814; doi:10.1111/mpp.13365)
Supplement: Supplementary file 13 — FILE S1 Supplementary methods. Details of bioinformatics methods used in this study [file MPP-24-1220-s005.docx]

**Supplementary Methods**

Details of bioinformatics methods used in this study.

**Whole genome resequencing analysis**

Sequencing reads were trimmed with fastp (S. Chen et al., 2018) using the parameters ‘--cut_tail --cut_tail_mean_quality=20 --detect_adapter_for_pe --length_required=75’, and aligned with the IPO323 genome with Bowtie 2 version 2.3.4.1 using --local mode (Langmead & Salzberg, 2012). Single nucleotide polymorphisms (SNPs) and indels were detected in the genome sequences via a Galaxy pipeline (https://usegalaxy.org/; Supplementary Methods), using FreeBayes for variant calling (Garrison & Marth, 2012). Polymorphisms were validated by inspection using the Integrative Genomics Viewer (IGV; Thorvaldsdóttir et al., 2013). The resulting polymorphisms were filtered to exclude those present in the isogenic IPO323 strain and identify those unique to the strains of interest.

The genomic location of T-DNA sequences was identified using discordant alignment of paired-end sequencing mates to chromosomal loci and T-DNA sequences. To achieve this, the relevant selective cassette was included in the reference genome as a separate chromosome. Alignments were carried out using the Bowtie 2 parameters --local, and -k 2. These parameters allow reads which arise from the junction between T-DNA sequences and their insertion sites to align to both of these regions. T-DNA insertion sites were determined using the mate location of reads aligned to the ends of heterologous T-DNA sequences and flanking regions used in the transformation vector.

***Z. tritici* alignments and differential expression analysis**

The *Z. tritici* IPO323 reference genome was indexed with genes annotated using the recent annotation from Rothamsted Research (King et al., 2017); https://figshare.com/articles/dataset/Zym_tritici_RRes_v4_0_RK_public_gff/4753708/1). Reads were trimmed to remove sequencing adapters and low quality bases using fastp (Chen et al., 2018), using the parameters ‘--cut_tail --cut_tail_mean_quality=20 --detect_adapter_for_pe --length_required=75’. Trimmed reads were aligned to the genome and gene counts calculated with STAR (Dobin et al., 2013), using the default parameters and set to quant mode --quantMode GeneCounts. Normalisation of gene counts and differential expression analysis was carried out using DESeq2 (Love et al., 2014). Sample clustering was assessed by principal component analysis using the *plotPCA* function on count data transformed with the *rlog* function, which accounts for sequencing depth and differences in variance between genes due to expression level (Love et al., 2014). Sets of genes which were differentially expressed (DE) between each mutant strain and the wild type at each time point were identified using the *results* function. Furthermore, sets of genes which were DE between 6 and 9 dpi for each strain were also identified. Wald test *p*-values were adjusted for multiple testing using the Benjamin–Hochberg (BH) correction method, and genes were deemed as DE when *padj*<0.01.

Plots displaying expression of individual genes across samples represent the mean of normalised gene counts from DESeq2. Heat maps representing the expression levels of different gene sets were generated with the *pheatmap* function. Heat maps represent log2 fold-changes compared to the mean of normalised counts calculated by the *rlog* function.

**Wheat alignments and differential expression analysis**

The *Triticum aestivum* (cv. Chinese Spring) wheat genome assembly and annotation from the International Wheat Genome Sequencing Consortium (IWGSC, 2018) was downloaded from Ensembl Plants (http://plants.ensembl.org/Triticum_aestivum). RSEM was chosen for wheat transcript quantification as it factors in the high number of multi-mapped reads, which result from the polyploidy of the wheat genome, using an expectation maximization (EM) algorithm (Deschamps-Francoeur et al., 2020; Li & Dewey, 2011). The reference genome was indexed with the RSEM *rsem-prepare-reference*, using the parameters --star and --star-sjdboverhang 149 to build indices for alignment with STAR (Dobin et al., 2013). Trimmed reads were then aligned to this reference and expected counts calculated using *rsem-calculate-expression*, specifying the parameters --star and --paired-end. Expected gene-level counts were filtered using the *filterByExpr* function before normalisation factors were calculated using the trimmed mean of M-values (TMM) method in EdgeR (Robinson et al., 2010; Robinson & Oshlack, 2010). The data was then transformed using the *voom* function, linear models were fitted to each gene using the *lmfit* function and empirical Bayes moderation of the standard errors was carried out using the *eBayes* function, all within the limma package (Ritchie et al., 2015). Genes were identified as DE between treatments using the moderated *t*-test within the *topTable* function, testing whether the log2-fold-change values for a particular contrast differ from 0 at a BH-corrected *padj*-threshold of 0.01.

Visualisation of expression of individual genes between samples was done by extracting the counts per million (CPM) values and plotting the mean CPM across each treatment. Heat maps were generated for gene sets using log-CPM values calculated using the *cpm* function in EdgeR.

Chen, S., Zhou, Y., Chen, Y. & Gu, J. (2018) Fastp: An ultra-fast all-in-one FASTQ preprocessor. *Bioinformatics*, 34, i884–i890.

Deschamps-Francoeur, G., Simoneau, J. & Scott, M.S. (2020) Handling multi-mapped reads in RNA-seq. *Computational and Structural Biotechnology Journal*, 18, 1569–1576.

Dobin, A., Davis, C.A., Schlesinger, F., Drenkow, J., Zaleski, C., Jha, S., et al. (2013) STAR: Ultrafast universal RNA-seq aligner. *Bioinformatics*, 29, 15–21.

Garrison, E. & Marth, G. (2012) Haplotype-based variant detection from short-read sequencing. *ArXiv12073907 Q-Bio*.

International Wheat Genome Sequencing Consortium (IWGSC) (2018) Shifting the limits in wheat research and breeding using a fully annotated reference genome. *Science*, **361**, eaar7191.

King, R., Urban, M., Lauder, R.P., Hawkins, N., Evans, M., Plummer, A., et al. (2017) A conserved fungal glycosyltransferase facilitates pathogenesis of plants by enabling hyphal growth on solid surfaces. *PLoS Pathogens*, 13, e1006672.

Langmead, B. & Salzberg, S.L. (2012) Fast gapped-read alignment with Bowtie 2. *Nature Methods*, 9, 357–359.

Li, B. & Dewey, C.N. (2011) RSEM: accurate transcript quantification from RNA-Seq data with or without a reference genome. *BMC Bioinformatics*, 12, 323.

Love, M.I., Huber, W. & Anders, S. (2014) Moderated estimation of fold change and dispersion for RNA-seq data with DESeq2. *Genome Biology*, 15, 550.

Ritchie, M.E., Phipson, B., Wu, D., Hu, Y., Law, C.W., Shi, W., et al. (2015) Limma powers differential expression analyses for RNA-sequencing and microarray studies. *Nucleic Acids Research*, 43, e47.

Robinson, M.D., McCarthy, D.J. & Smyth, G.K. (2010) edgeR: A Bioconductor package for differential expression analysis of digital gene expression data. *Bioinformatics*, 26, 139–140.

Robinson, M.D. & Oshlack, A. (2010) A scaling normalization method for differential expression analysis of RNA-seq data. *Genome Biology*, 11, R25.

Thorvaldsdóttir, H., Robinson, J.T. & Mesirov, J.P. (2013) Integrative Genomics Viewer (IGV): high-performance genomics data visualization and exploration. *Briefings in Bioinformatics*, 14, 178–192.
